# Clinical utility of liquid biopsy and integrative genomic profiling in early-stage and oligometastatic cancer patients treated with radiotherapy

**DOI:** 10.1038/s41416-022-02102-z

**Published:** 2022-12-22

**Authors:** Guadalupe A. Cifuentes, Adrián Santiago, Lucía Méndez Blanco, María Fueyo, Esther López Martínez, Raquel Soria, Irene Martín López, Pepa Cucarella Beltrán, Pablo Pardo-Coto, David Rodriguez-Rubi, Karla Urquilla, Noelia S. Durán, Rebeca Álvarez, Claudia G. Lago, Andrea Otero, Marta Diñeiro, Raquel Capín, Juan Cadiñanos, Rubén Cabanillas

**Affiliations:** 1 Laboratorio de Medicina Molecular, Instituto de Medicina Oncológica y Molecular de Asturias (IMOMA), Oviedo, Asturias Spain; 2 Servicio de Oncología Radioterápica, Instituto de Medicina Oncológica y Molecular de Asturias (IMOMA, Oviedo, Asturias Spain; 3 Oncología Médica, Centro Médico de Asturias, Oviedo, Asturias Spain; 4 Área de Medicina de Precisión, Instituto de Medicina Oncológica y Molecular de Asturias (IMOMA), Oviedo, Asturias Spain; 5 Present Address: Cabanillas Precision Consulting (CPC), Zurich, Switzerland

**Keywords:** Tumour biomarkers, Cancer genomics, Cancer genetics, Predictive markers, Prognostic markers

## Abstract

**Background:**

Liquid biopsy and Integrative Genomic Profiling (IGP) are yet to be implemented into routine Radiation Oncology. Here we assess the utility of germline, tumour and circulating cell-free DNA-based genomic analyses for the clinical management of early-stage and oligometastatic cancer patients treated by precision radiotherapy.

**Methods:**

We performed germline, tissue- and liquid biopsy NGS panels on 50 early-stage/oligometastatic cancer patients undergoing radiotherapy. We also monitored ctDNA variants in serial liquid biopsies collected during radiotherapy and follow-up and evaluated the clinical utility of such comprehensive approach.

**Results:**

The integration of different genomic studies revealed that only 1/3 of the liquid biopsy variants are of tumour origin. Altogether, 55 tumour variants (affecting 3/4 of the patients) were considered potentially actionable (for treatment and prognosis), whereas potential follow-up biomarkers were identified in all cases. Germline cancer-predisposing variants were present in three patients, which would have not been eligible for hereditary cancer testing according to clinical guidelines. The presence of detectable ctDNA variants before radiotherapy was associated with progression-free survival both in oligometastatic patients and in those with early-stage.

**Conclusions:**

IGP provides both valuable and actionable information for personalised decision-making in Radiation Oncology.

## Introduction

Somatic and germline Integrative Genomic Profiling (IGP) and circulating tumour DNA (ctDNA) characterisation (liquid biopsy) have become a cornerstone of Precision Oncology. Translating Precision Oncology into clinical practice entails disease prevention, accurate diagnoses, personalised treatments, and individualised follow-ups for patients and their families [1, 2]. Moreover, tumour-agnostic trials, based on the recruitment of patients with specific molecular alterations independently of their histology, rely strongly on accurate and broad genomic characterisation [3]. And although only a couple of biomarker-driven tissue-agnostic indications have been approved to date, they represent a significant milestone for targeted therapies in Precision Medicine [4–6].

Germline testing plays a major role in the management of cancer patients. The discovery of a predisposing genomic variant is a game-changer for the prevention of secondary malignancies and familiar genetic counselling [7, 8]. Testing only patients with suggestive family histories—as defined by clinical practice guidelines—may miss up to 50% of patients with an actionable pathogenic germline variant [7]. Moreover, parallel somatic and germline testing can distinguish germline and clonal haematopoiesis (CH)-derived alterations from those derived from the tumour [9, 10].

While introducing IGP in clinical practice, tissue samples are not always technically feasible or can imply risks. Also, they represent a static picture of the evolving genomic scenario of a tumour, unable to assess its genomic heterogeneity [11–15] accurately. Therefore, while embracing IGP, ctDNA provides a useful tool whose full potential remains to be uncovered. Usually referred to as liquid biopsy, ctDNA represents the fraction of cell-free DNA (cfDNA) derived from the tumour in cancer patients. While blood cfDNA concentration ([cfDNA]) has been described to be higher in cancer patients than in healthy controls, it can vary substantially between patients [16–18]. Many factors can affect blood [cfDNA]: physical activity, trauma, diet or other health conditions [19]. Specifically, ctDNA levels are also affected by disease load, tumour location and vascularisation, as well as cellular turnover and cancer treatments [17, 20–22]. Despite their limitations, owing to their minimally invasive nature, ctDNA and [cfDNA] stand as compelling options for diagnosis and longitudinal assessment of the tumour throughout the disease course.

Many studies have shown a good correlation between tissue and liquid biopsies in advanced-stage patients [22–27]. However, in cases with low tumour volume, such as early stages or oligometastatic disease, the correlation remains ill-defined. Besides, in everyday clinical practice, IGP has been more widely used for advanced-stage cancer patients, often relapsed or refractory to previous standard cytotoxic treatments, exclusively with the aim to select a new therapeutic agent. These patients usually have a poor prognosis and short-life expectancy, with an inherently negative effect on the assessment of IGP utility, undermining benefits such as genetic counselling or follow-up. Even more, regardless of the high sensitivity of novel technologies for ctDNA analysis [28–30], since ctDNA fraction is lower in earlier stages of the disease, ctDNA characterisation remains challenging in patients with low tumour burden [17, 31].

Most studies have focused on the correlation between chemotherapy or targeted systemic treatments and ctDNA, while the role of radiotherapy (RT) on ctDNA kinetics is characterised by many unknowns. This knowledge gap has to be addressed since it is estimated that about 50% of cancer patients receive some form of RT in the course of their disease [32]. In the last decades, technological advances have meant a revolution in the accuracy and safety of these treatments. Compared to conventionally fractionated radiotherapy (CFRT), modern techniques such as stereotactic body radiotherapy (SBRT) can deliver high doses of radiation to a target with high precision, using a single dose/small number of fractions. Nowadays, SBRT is a common tool in the management of early-stage lung cancer and oligometastatic disease [33, 34]. As surgery is contraindicated for most patients treated with SBRT, tissue biopsies are also usually inadvisable or limited. Therefore, liquid biopsies stand as a great alternative for molecular analysis in these patients. Furthermore, different mechanisms of cell death are proposed to be caused by RT, but the responsible molecular processes remain unclear [35–39]. The study of cfDNA/ctDNA can be a good approach to understand the mechanisms and dynamics of cell death caused by RT, and could be a key to improving and personalising the treatments.

Therefore, there is an urgent need to deepen the role of liquid biopsies in early-stage or oligometastatic patients, especially when receiving curative or radical-palliative treatments different from palliative systemic chemotherapy. With this scenario, the present work was designed as a real-world prospective study aimed to assess the actionability of germline and both tissue biopsy and liquid biopsy-based somatic IGP in a cohort of 50 early-stage/oligometastatic patients undergoing precision RT. In addition, we investigate the utility of liquid biopsy as a novel biomarker in radiation oncology, with the main purpose of contributing to the translation of Precision Oncology into clinical practice.

## Results

### Cohort description

**Fig. 1:**
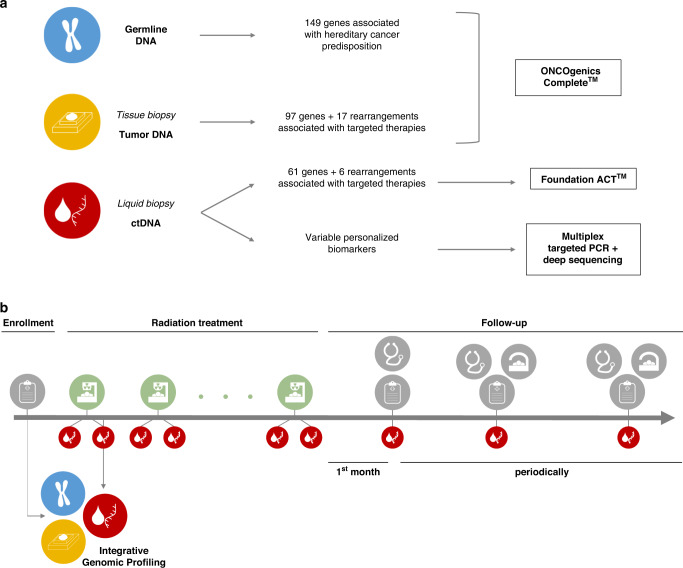
Integrative Genomic Profiling (IGP) strategy and study design. **a** IGP strategy: three different specimens were analysed using three NGS panels for genomic characterisation in each patient; the target regions are summarised (detailed in Supplementary Methods). Selected personalised biomarkers were later interrogated in serial liquid biopsies using a targeted method. **b** Study design: after the recruitment of candidate patients who fulfilled the inclusion criteria, specimens for IGP were collected. Once RT treatment was initiated, liquid biopsies were performed before and after each session and, also, periodically during the follow-up for further targeted ctDNA analysis. In addition, all the available clinical information, as medical records, patient status and results from diagnostic imaging tests, was gathered.

The cohort consisted of 50 patients. The main demographics and clinical characteristics are summarised in Table 1 (detailed in Supplementary Table 1). 18/50 patients had clinical stage I disease (36%), and 32/50 had stage IV disease (64%), 31 oligometastatic and 1 high-grade glioblastoma multiforme. 18/50 patients had early-stage non-metastatic lung cancer (group “ES”; 36.0%); 18/50 had the oligometastatic disease (single or multiple metastases) without active malignant disease out of the radiation field (group “OMT”; 36.0%); and 14/50 had the oligometastatic disease (single or multiple metastases) with active malignant disease out of the radiation field (group “OMT + ”; 28.0%). From OMT and OMT + groups, 57.6% presented oligometastases in the lung. 45 patients (90%) were treated with hypofractionated radiation therapy (HFRT) and 5 (10%) were treated with CFRT. RT dose-fractionation schedules are detailed in Table 2. The mean follow-up duration was 9 months (ranging 0–23 months). 46/50 patients underwent full IGP (Fig. 1a): in four patients, the tumour (3 cases) or liquid biopsy samples (1 case) did not fulfil requirements. For RT monitoring, the full schedule of blood draws was completed in 44. Figure 1b illustrates the study design.

**Table 1:** Patient’s demographics and clinical characteristics.

Sex			
Male	34		
Female	16		
**Age at treatment median (range):**	68 (41–87)		
**Smoking history**			
Smoker	13		
Former smoker	27		
Non-smoker	10		
**Primary tumour**			
Lung	Non-small cell lung cancer	Adenocarcinoma	14
		Squamous cell carcinoma	10
		Poorly differentiated	3
	Small cell carcinoma	4	
Colon	Adenocarcinoma	7	
Uterus	Carcinosarcoma	1	
	Leiomyosarcoma	1	
	Squamous cell carcinoma	1	
Pharynx	Squamous cell carcinoma	2	
Bladder	Adenocarcinoma	1	
	High-grade transitional cell carcinoma	1	
Skin	Melanoma	1	
	Pilomatrix carcinoma	1	
Brain	High-grade glioblastoma	1	
Pancreas	Ductal adenocarcinoma	1	
Kidney	Clear cell carcinoma	1	
**Stage**			
I	18		
IV	32		
**Group**			
ES	18		
OMT	17		
OMT+	15		

Treatment				
RT type	RT subtype	Irradiated lesion	Primary tumour organ	
HFRT	SBRT	Lung primary	Lung	20
		Lung primary and metastasis	Lung	1
		Lung metastasis	Colon	5
			Uterus	2
			Lung	2
			Pharynx	1
			Skin	1
			Bladder	2
		Liver metastasis	Colon	1
			Kidney	1
			Lung	1
		Umbilical metastasis	Pancreas	1
		Soft tissues neck metastasis	Lung	1
			Uterus	1
	RS	Brain metastasis	Lung	1
			Skin	1
	HRS	Brain metastasis	Lung	2
			Colon	1
CFRT	—	Lung primary	Lung	1
		Lung primary and metastasis	Lung	1
		Soft tissues neck metastasis	Lung	1
		Primary and metastases	Pharynx	1
		Brain primary	Brain	1

**Table 2:** Radiotherapy dose-fractionation schedules.

Patient #	Clinical data				Treatment characteristics							
	Primary tumour—organ	Primary tumour—histology	Irradiated lesion (number of lesions, if multiple)	Irradiated organ	RT type	RT subtype	Number of fractions	Total dose (Gy)	Dose per fraction (Gy)	Post-RT session liquid biopsy timelag (minutes)	Varaition post-RT session liquid biopsy timelag (minutes)^a^	Liquid biopsy panel
1	Lung	NSCLC (adenocarcinoma)	Primary	Lung	HFRT	SBRT	5	55	11	15	15	15 min after session 1
2	Lung	NSCLC (adenocarcinoma)	Primary	Lung	HFRT	SBRT	5	60	12	15	15 / (30)	15 min after session 1
3	Lung	NSCLC (poorly differentiated carcinoma)	Primary	Lung	HFRT	SBRT	5	60	12	15	15 / (30)	30 min after session 5
4	Lung	NSCLC (squamous cell carcinoma)	Primary	Lung	HFRT	SBRT	5	55	11	30	(15) / 30	30 min after session 1
5	Skin	Pilomatrix carcinoma	Metastasis	Lung	HFRT	SBRT	5	55	11	30	30	30 min after session 1
6	Colon	Adenocarcinoma	Metastasis	Liver	HFRT	SBRT	5	50	10	30	30	30 min after session 2
7	Uterus	Leiomysosarcoma	Metastasis	Soft tissues neck	HFRT	SBRT	5	30	6	30	30	30 min after session 1
8	Uterus	Squamous cell carcinoma	Metastasis	Lung	HFRT	SBRT	5	60	12	30	30	30 min after session 1
9	Kidney	Clear cell carcinoma	Metastasis	Liver	HFRT	SBRT	5	50	10	30	30	30 min after session 1
10	Uterus	Carcinosarcoma	Metastasis	Lung	HFRT	SBRT	5	60	12	30	30	30 min after session 1
11	Lung	NSCLC (squamous cell carcinoma)	Metastasis	Lung	HFRT	SBRT	5	50	10	30	30	30 min after session 1
12	Bladder	Adenocarcinoma	Metastasis	Lung	HFRT	SBRT	5	55	11	30	30	30 min after session 1
13	Lung	NSCLC (squamous cell carcinoma)	Primary	Lung	HFRT	SBRT	5	60	12	30	30	30 min after session 1
14	Lung	NSCLC (squamous cell carcinoma)	Primary	Lung	HFRT	SBRT	5	55	11	30	30	30 min after session 1
15	Lung	NSCLC (adenocarcinoma)	Primary	Lung	HFRT	SBRT	5	55	11	30	30	30 min after session 1
16	Lung	SCLC	Metastasis	Liver	HFRT	SBRT	5	45	9	30	30	30 min after session 2
17	Colon	Adenocarcinoma	Metastases (5)	Lung	HFRT	SBRT	5	55	11	30	30	30 min after session 1
18	Lung	NSCLC (adenocarcinoma)	Primary and lung metastasis	Lung	HFRT	SBRT	5	55	11	30	30	30 min after session 4
19	Lung	NSCLC (poorly differentiated carcinoma)	Primary	Lung	HFRT	SBRT	5	55	11	30	30 / (45)	30 min after session 1
20	Lung	NSCLC (adenocarcinoma)	Primary	Lung	HFRT	SBRT	5	40	8	30	30 / (45)	30 min after session 1
21	Pancreas	Ductal adenocarcinoma	Metastasis	Navel	HFRT	SBRT	5	30	6	30	30 / (45)	45 min after session 1
22	Lung	NSCLC (squamous cell carcinoma)	Primary	Lung	HFRT	SBRT	5	60	12	60	60 + multi	60 min after session 1
23	Lung	SCLC	Primary	Lung	HFRT	SBRT	5	50	10	60	60	60 min after session 1
24	Lung	NSCLC (adenocarcinoma)	Primary	Lung	HFRT	SBRT	5	60	12	60	60	60 min after session 1
25	Lung	NSCLC (adenocarcinoma)	Primary	Lung	HFRT	SBRT	5	60	12	60	60	60 min after session 1
26	Lung	NSCLC (squamous cell carcinoma)	Primary	Lung	HFRT	SBRT	5	50	10	60	60	60 min after session 1
27	Bladder	High-grade transitional cell carcinoma	Metastases (2)	Lung	HFRT	SBRT	8 / 5	60 / 55	7.5 / 11	60	30	30 min after session 1
28	Colon	Adenocarcinoma	Metastases (5)	Lung	HFRT	SBRT	8 / 8 / 8 / 8 / 5	60 / 60 / 60 / 60 / 60	7.5 / 7.5 / 7.5 / 7.5 / 12	60	60	60 min after session 1
29	Lung	NSCLC (adenocarcinoma)	Metastasis	Brain	HFRT	HRS	3	27	9	30	multi	30 min after session 1
30	Lung	NSCLC (poorly differentiated carcinoma)	Primary	Lung	HFRT	SBRT	8	60	7.5	30	30 + multi	30 min after session 1
31	Lung	NSCLC (squamous cell carcinoma)	Primary	Lung	HFRT	SBRT	8	60	7.5	30	30 + multi	30 min after session 1
32	Lung	NSCLC (adenocarcinoma)	Primary	Lung	HFRT	SBRT	8	60	7.5	30	30	30 min after session 1
33	Lung	NSCLC (adenocarcinoma)	Primary	Lung	HFRT	SBRT	8	60	7.5	30	30	30 min after session 1
34	Pharynx	Squamous cell carcinoma	Metastases (2)	Lung	HFRT	SBRT	8	60	7.5	30	30	30 min after session 1
35	Colon	Adenocarcinoma	Metastasis	Lung	HFRT	SBRT	8	60	7.5	30	30 / (45)	30 min after session 1
36	Lung	NSCLC (adenocarcinoma)	Metastasis	Soft tissues neck	HFRT	SBRT	3	27	9	30	30	30 min after session 1
37	Lung	NSCLC (squamous cell carcinoma)	Primary	Lung	HFRT	SBRT	3	54	18	30	30	30 min after session 1
38	Lung	NSCLC (adenocarcinoma)	Metastasis	Lung	HFRT	SBRT	3	60	20	30	30	30 min after session 1
39	Colon	Adenocarcinoma	Metastases (2)	Lung	HFRT	SBRT	3	60	20	60	60	60 min after session 1
40	Lung	NSCLC (adenocarcinoma)	Primary	Lung	HFRT	SBRT	3	54	18	60	(45) / 60	45 min after session 1
41	Colon	Adenocarcinoma	Metastasis	Lung	HFRT	SBRT	3	54	18	15	15	15 min after session 1
42	Colon	Adenocarcinoma	Metastases (2)	Brain	HFRT	RS / HRS	1 / 3	20 / 27	20 / 9	30	30	30 min after session 2
43	Lung	NSCLC (squamous cell carcinoma)	Metastases (3)	Brain	HFRT	RS	1	24	24	30	30	30 min after session 1
44	Skin	Melanoma	Metastasis	Brain	HFRT	RS	1	15	15	30	30	30 min after session 1
45	Lung	NSCLC (squamous cell carcinoma)	Metastases (4)	Brain	HFRT	HRS	10 / 10	30 / 40	3 / 4	30	30	30 min after session 1
46	Brain	High-grade glioblastoma	Primary	Brain	CFRT	.	30	60	2	60	(45) / 60	45 min after session 21
47	Pharynx	Squamous cell carcinoma	Primary and pharynx metastases	Pharynx	CFRT	.	35	70	2	60	60	Failed
48	Lung	SCLC	Primary and lung metastasis	Lung	CFRT	.	35	70	2	30	30 / (45)	30 min after session 10
49	Lung	NSCLC (adenocarcinoma)	Metastases	Soft tissues neck	CFRT	.	33	69.96	2.12	30	30 / (45)	30 min after session 5
50	Lung	SCLC	Primary	Lung	CFRT	.	30	60	2	30	30	1 week before session 1

*NA* not applicable, *NSCLC* non-small cell lung cancer, *SCLC* small cell lung cancer, *RT* radiotherapy, *HFRT* hypofractionated radiation therapy, *SBRT* stereotactic body radiotherapy, *RS* radiosurgery, *HRS* hypofractionated radiosurgery, *CFRT* conventionally fractionated image-guided radiotherapy.

^a^For those patients with multiple values, different timelags in different sessions; multi: multiple post-RT liquid biopsies

### Findings of the different IGP tests

#### The importance of germline sequencing for variant origin elucidation

Liquid biopsy panels are frequently performed with no germline sample tested concurrently. We hypothesised that analysing germline samples would improve the identification of real tumour variants and variant origin elucidation.

**Fig. 2:**
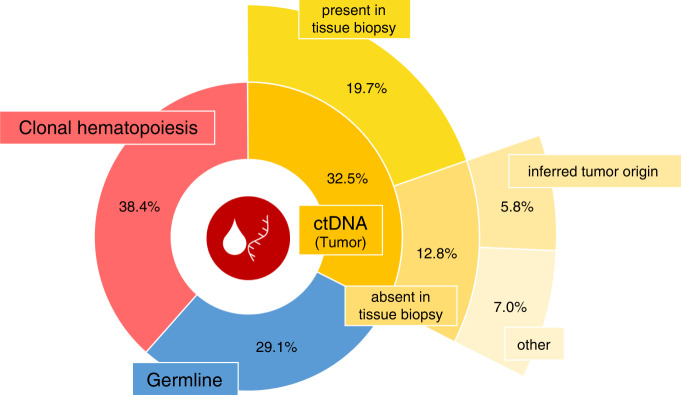
Origin of the variants detected by liquid biopsy test. By comparing the results obtained by the liquid biopsy panel to those from the other genomic panels, the origin of most of the identified variants was clarified. 29.1% (25/86) of the variants have germline origin (blue), 38.4% (33/86) were derived from clonal haematopoiesis (red), and the remaining 32.6% (28/86) were considered real ctDNA variants, potentially derived from the tumour (yellow). Regarding those tumour ctDNA variants, 17 were present in the tissue biopsy (60.7% of ctDNA variants, 19.8% of total) and 11 were not (39.3% of ctDNA variants, 12.8% of total). Of those absent in the tissue biopsy, other 5 variants were assumed to derive from the tumour in the light of serial liquid biopsies results (17.9% of ctDNA variants, 5.8% of total) whereas the suspected tumour origin of the other 6 (21.4% of ctDNA variants, 7.0% of total) could not be ascertained.

In our cohort, 29.1% of the 86 variants detected by the liquid biopsy panel (from 35 patients, with at least one variant) had germline origin and, thus, were not eligible as follow-up biomarkers (Fig. 2, all variants are included in Supplementary Table 2). They had an average mutation allelic frequency (AF) of 48.93% in liquid biopsy panel testing (range 24.09–53.07%). Of note, one *TP53* variant confirmed to have tumour origin had a similar AF (47.28%), so, in the absence of germline testing, it could have been erroneously inferred as germline-derived. On the other hand, a direct search of the remaining liquid biopsy variants both on the results of germline sequencing and by targeted PCR and deep sequencing, revealed that 38.4% of the variants initially identified by the liquid biopsy panel have a hematopoietic origin. As a result, only 28 (32.5%) of the variants initially identified by the liquid biopsy panel are tumour-specific (henceforth referred to as “ctDNA variants”). (Fig. 2). Hence, germline testing did improve the precision of ctDNA variant identification.

#### Characterisation and exploration of clonal haematopoiesis in the cohort

As clonal hematopoiesis (CH) was the origin of most variants initially identified by the liquid biopsy test, we set to explore potential associations between this phenomenon and cancer-related variables. CH-related variants showed an average AF of 0.73% (ranging 0.11–5.45%) in the liquid biopsy panel. The observed 33 CH-related variants affected 18 different genes, being *TP53* (10), *NF1* (3), *PTEN* (3), *BRCA2* (2) and *JAK2* (2) the most recurrent. *TP53* and *JAK2* CH-related variants have been associated with clonal hematopoiesis of indeterminate potential (CHIP) [40]. All the CH-related variants were detected on cfDNA during treatment and follow-up. Particularly, patients #14 and #24 carry the *JAK2* c.1849G > T; p.Val617Phe variant, associated with polycythaemia vera, essential thrombocythaemia and primary myelofibrosis [41, 42]. This variant mostly maintained a VAF under 2%, the threshold for a CHIP variant [43], and these patients had no history of myeloproliferative disorders. Age was significantly higher in patients carrying CH-related variants than in non-carriers (*p*-value = 0.00001, Student’s *t*-test). No association was found between the presence of CH-variants and prior cytotoxic treatments nor smoking habits in our cohort (*p*-value > 0.05, Chi-squared test). Thus, apart from age (a known risk factor for CH), none of the explored variables was associated with CH in our cohort.

#### Comparative mutational landscape of ES, OMT and OMT + patients

A total of 223 variants identified by the tissue biopsy panel were confirmed tumour variants. At least one tumour variant was identified in every patient, with a mean of 4.8 variants/sample. A total of 28 confirmed tumour variants were detected by the liquid biopsy panel. With a median of 1 ctDNA variant per case (range 1–5), at least one tumour variant was present in 41.3% of the patients (19/46); the average AF of these ctDNA variants was 2.50% (range 0.1–47.28%).

**Fig. 3:**
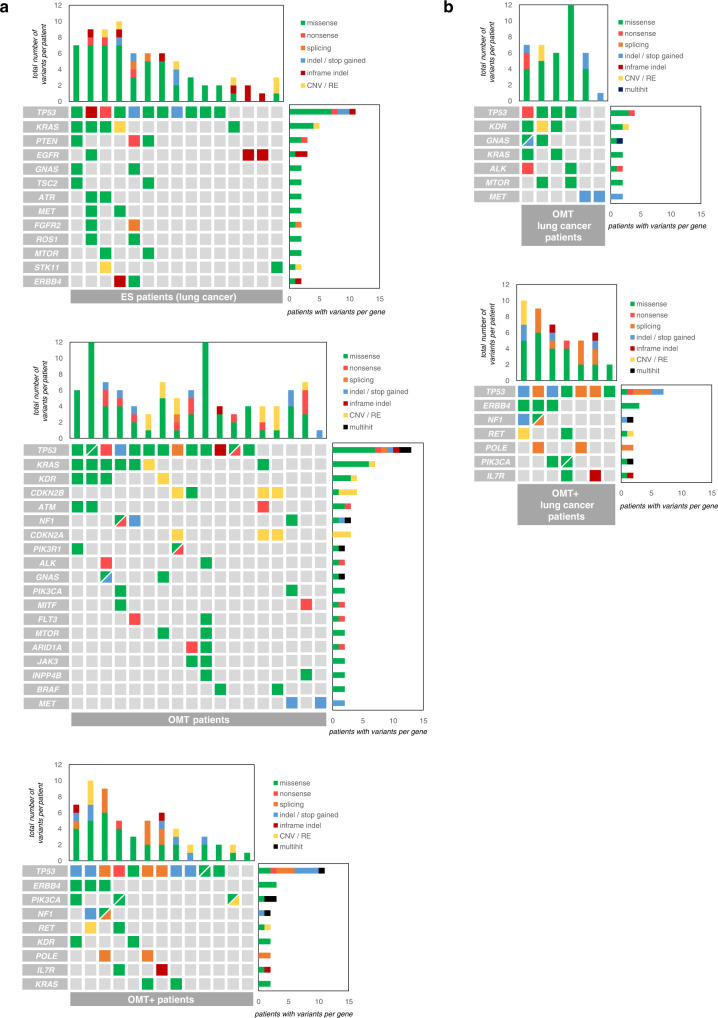
Oncoplot of the most frequently altered genes in the cohort. **a** ES, OMT and OMT + patients represented in separate oncoplots (all histologies included) (in Supplementary Fig. 1A all these patients are plotted together). **b** OMT and OMT + lung cancer patients plotted separately (all ES patients from Fig. 3a corresponded to lung cancer), (in Supplementary Fig. 1B all these patients are plotted together). Only confirmed somatic driver variants and variants of unknown significance (VUS) identified by the tissue and/or the liquid biopsy panel tests are shown. The different types of genetic alterations are represented by colours. All genes mutated in at least two patients are depicted. Columns represent the 46 patients with full IGP, clustered per clinical groups. The full list of all variants considered for this figure is included in Supplementary table 2. CNV copy number variations, RE rearrangements.

We looked at the distribution of tumour mutations (found in the tumour tissue and/or liquid biopsies) within our different clinical groups (ES, OMT and OMT+). The mutational landscape of all patients revealed that *TP53* is the most frequently mutated cancer gene in the whole cohort and in all the subgroups, which otherwise show diverse mutational profiles (Fig. 3a, Supplementary Fig. 1A and Supplementary Table 3). As our cohort comprised patients with cancers of different primary sites, we focused on 28 patients with lung cancer (Fig. 3b and Supplementary Fig. 1B). Their mutational profile showed large similarities to that of 916 lung cancer patients from the Clinical Proteomic Tumour Analysis Consortium 3 (CPTAC-3) (Supplementary Fig. 1C). For example, 9 out of 15 top mutated genes are coincident between both groups, or alterations in *PIK3CA* and/or *NF1* tend to be mutually exclusive with those in *KRAS* (Supplementary Fig. 1B and Supplementary Fig. 1C*)*. These results indicate that our lung cancer subset resembles the wider lung cancer population.

#### Concordance between tumour variants detected by tissue and liquid biopsies

**Fig. 4:**
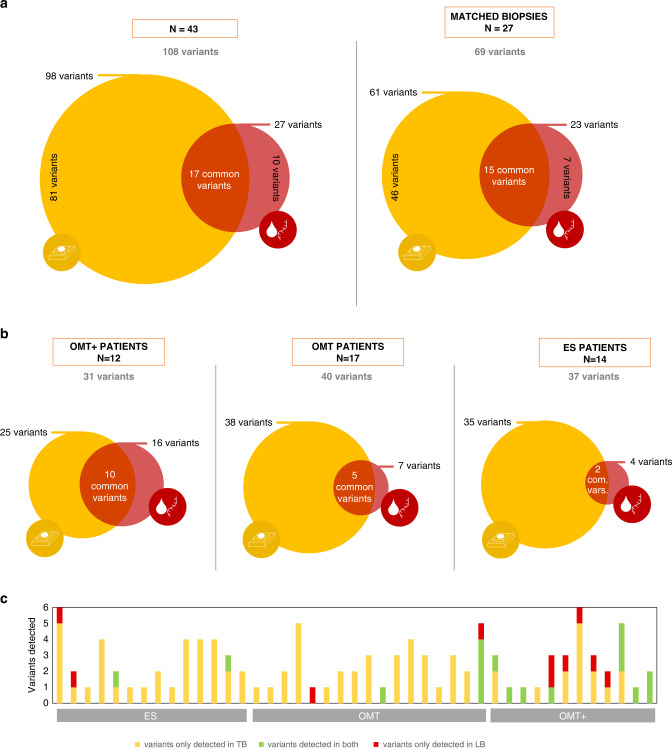
Concordance between tissue biopsy and liquid biopsy tests. **a** On the left, out of 108 total variants identified, 98 (90.7%) were detected by the tissue panel (yellow) and 27 (25.9%) by the liquid biopsy panel (red), with 17 being present in both tests (15.7%, the intersection). 63.0% (17/27) of the variants detected by the liquid biopsy panel were also found in the tissue, while only 17.3% (17/98) of the tissue variants were found in the liquid biopsy panel. 3 patients are not included due to the lack of variants in the genomic tests. Tissue and liquid biopsies have a median delay time of 118 days (range 9–2112 days). Restricting to the 27 cases with matched biopsies (diagram on the right), where the median delay time is 60 days (ranging 9–518 days), the concordance rate was higher, 21.7% (15/69) of all variants were identified by both tests. 65.2% (15/23) of the variants detected by the liquid biopsy panel were also found in the tissue, while only 24.6% (15/61) of the tissue variants were found by liquid biopsy panel. **b** Venn diagram for liquid biopsy panel and tissue panel concordances plotted separately according to clinical stage group. **c** Stacked column chart showing the variant concordance between the panel test for each patient. In all panels, only genomic regions covered by both platforms are considered. Germline and CH-derived variants are excluded for comparison.

As the tumour burden of our patients (with early-stage and oligometastatic cancer) is lower than that of cohorts with advanced disease, we hypothesised that concordance would likely be lower, too [22–24, 26]. Seventeen of the 28 ctDNA variants detected by liquid biopsy panel test were present in the tissue biopsy, and 11 were not (Fig. 2). 15.7% of all the somatic variants identified were detected by both panels (17/108) (Fig. 4), as expected, lower than reported in advanced disease cohorts, with concordance rates around 80% [22–24, 26]. Concordance increased to 21.7% (15/69) in the 27 cases with matched biopsies (where the tested tissue biopsy corresponded to the irradiated lesion) (Fig. 4a) and was highest in those patients with the most advanced disease (OMT + : 32.3%; 10/31) (Fig. 4b, c). Similar findings were observed when restricting the analysis to lung cancer patients (Supplementary Fig. 2).

### Clinical utility of IGP

We then set to evaluate the ability of our integrative approach to identify clinically relevant variants. Somatic and germline results concerning targeted therapies, prognosis, biomarker discovery or genetic counselling obtained by IGP are shown in Table 3.

**Table 3:** Actionability provided by germline and somatic tissue and liquid biopsy-based.

Patient #	Diagnosis	Treatment				Follow-up		Other types of actionability	
		ESCAT tier	Tissue biopsy	Liquid biopsy	Clinical trials	Tissue biopsy	Liquid biopsy	Tissue biopsy	Liquid biopsy
1	NSCLC (adenocarcinoma)	IV-A	*KRAS* p.Gly12Val cobimetinib, binimetinib, trametinib	—	NCT03170206, NCT04735068, NCT03948763	ATR p.Leu341Phe / EGFR p.Gly517Cys / GNA11 p.Glu245Lys / IGF1R p.Glu979* / KRAS p.Gly12Val / MET p.Asp1222Asn / ROS1 p.Met1118Ile / TP53 p.Phe341_Arg342delinsLeu	*FGFR2* p.Glu777Lys	KRAS p.Gly12Val PROGNOSIS	—
2	NSCLC (adenocarcinoma)	I-A	*EGFR* p.Glu746_Ala750del afatinib, dacomitinib, erlotinib, erlotinib + ramucirumab, gefitinib, osimertinib	—	NA	*EGFR* p.Glu746_Ala750del	*BRAF* N486_T491>K	—	—
		III-B	—	BRAF N486_T491>K dabrafenib + trametinib	NCT04620330				
3	NSCLC (poorly differentiated carcinoma)	—	—	—	—	BAP1 p.Ala134Thr / JAK1 p.Ser1137Pro / TP53 p.Tyr220Cys	—	—	—
4	NSCLC (squamous cell carcinoma)	III-A	*PIK3CA* p.Glu542Lys alpelisib + fulvestrant (other tumour)		NCT04073680, NCT04774952	IL7R p.Lys395Arg / PIK3CA p.Glu110Lys / PIK3CA p.Glu542Lys / RET p.Pro1053Ser / TP53 p.Gln331*	PIK3CA p.Glu542Lys	*EXT1* p.Tyr119* GENETIC COUNSELLING	—
		III-A	*PIK3CA* p.Glu110Lys alpelisib + fulvestrant (other tumour)	—	NCT04073680, NCT04774952				
5	Pilomatrix carcinoma	III-A	*CTNNB1* p.Asp32Tyr everolimus + letrozole, imatinib, vinorelbina (other tumour)	—	—	CSF1R p.Arg579Trp / CTNNB1 p.Asp32Tyr / FGFR4 p.Arg606Gln / TP53 p.Arg248Trp	—	*BRIP1* p.Phe176Serfs*9 GENETIC COUNSELLING	—
		III-A	*BRIP1* p.Phe176Serfs*9 olaparib (other tumour)	—	NCT04171700				
6	Colon adenocarcinoma	III-A	*FBXW7* p.Arg465Cys temsirolimus, veliparib, sirolimus, everolimus, (other tumour)	—	—	FBXW7 p.Arg465Cys / KDR p.Gln268Lys / TP53 p.Arg273Cys	*TP53* p.Arg273Cys	—	—
7	Uterine leiomyosarcoma	—	—	—	—	*TP53* p.Pro27Leufs*17	*TP53* p.Pro27Leufs*17	—	—
8	Squamous cell carcinoma of the cervix	III-A	*PIK3CA* amplification N=6, *PIK3CA* p.Glu542Lys alpelisib + fulvestrant (other tumour)	-	NCT04073680, NCT04774952, NCT04836663	*PIK3CA* p.Glu542Lys	—	—	—
9	Clear cell renal cell carcinoma	—	—	—	—	*CSF3R* p.Asp172Asn	—	—	—
10	Uterine carcinosarcoma	III-A	*BRCA1* p.Gln1111Asnfs*5 niraparib, olaparib, rucaparib, talazoparib (other tumour)	—	NCT04171700, NCT03552471	PIK3R1 p.Arg358Pro / PIK3R1 p.Gln579* / TP53 c.993+1G>A	—	*BRCA1* p.Gln1111Asnfs*5 GENETIC COUNSELLING	—
		IV-A	*PIK3R1* p.Gln579* PI3K inhibitors, mTOR inhibitors, AKT inhibitors	—	NCT04836663				
12	Adenocarcinoma of the bladder	IV-A	*KRAS* amplification N=9 cobimetinib, binimetinib, trametinib	—	NCT03634982	*TP53* p.Cys275Arg	—	CCND1 amplification N = 7PROGNOSIS	—
13	NSCLC (squamous cell carcinoma)	III-A	*PTEN* p.Gly165Glu everolimus, tensirolimus (other tumours)	—	NCT04591431, NCT02465060	IGF2 p.Thr40Asn / JAK3 c.566+1G>T / MTOR p.Ala1971Val / PTEN p.Gly165Glu / TP53 p.Pro250Leu / TSC2 p.Arg344Met	—	—	—
14	NSCLC (squamous cell carcinoma)	III-A	*MAP2K1* p.Lys57Asn trametinib (other tumour)	—	NCT04488003	MAP2K1 p.Lys57Asn / TP53 p.Arg156Pro	*TP53* p.Arg156Pro	—	—
15	NSCLC (adenocarcinoma)	I-A	*KRAS* p.Gly12Cys sotorasib	—	NA	CCND3 p.Arg33Cys / KRAS p.Gly12Cys	—	KRAS p.Gly12Cys PROGNOSIS	—
16	SCLC	III-A	—	*PTEN* p.His61Arg, *PTEN* c.635-1G>C everolimus, tensirolimus (other tumours)	NCT04774952, NCT04591431	MTOR p.Gly1064Glu / IL7R p.Gly215Phe / ROS1 p.Ser1279Cysfs*51 / TP53 c.560-2A>G	PTEN p.His61Arg / PTEN c.635-1G>C / TP53 c.560-2A>G	—	—
17	Colon adenocarcinoma	III-A	*NF1* p.Gln786* selumetinib, trametinib (other tumour)	—	NCT02465060	KRAS p.Gly13Asp / MITF p.Arg406Gln / NF1 p.Gln786* / NF1 p.Ala1429Thr / PIK3CA p.Glu545Lys / TP53 p.Cys229Tyrfs*10	—	—	—
		III-A	*PIK3CA* p.Glu545Lys alpelisib + fulvestrant (other tumour)	—	NCT04073680, NCT04774952				
		IV-A / NA (resistance)	*KRAS* p.Gly13Asp cobimetinib, binimetinib, trametinib / anti-EGFR antibody (resistance)	—	NCT04117087, NCT03948763				
18	NSCLC (adenocarcinoma)	-—	—	—	—	FRS2 p.Asp505Valfs*? / GATA3 p.Ile351Ser / GATA3 p.Gly361Asp / MET p.Asn879Lysfs*23 / PIK3CA p.Asp258Tyr	*NF1* p.Ala1716Gly	—	—
19	NSCLC (poorly differentiated carcinoma)	—	—	—	—	FBXW7 p.Asp119Tyr / TP53 p.Val157Phe	—	—	—
20	NSCLC (adenocarcinoma)	—	—	—	—	*STK11* p.Cys132Arg	—	—	—
21	Pancreatic ductal adenocarcinoma	III-A	*ATM* p.Arg2993* olaparib (other tumour)	—	NCT03611868, NCT03742895	ATM p.Arg2993* / KRAS p.Gly12Arg	—	—	—
		IV-A	*KRAS* p.Gly12Arg cobimetinib, binimetinib, trametinib	—	NCT04348045, NCT04117087, NCT03948763		—	—	—
23	SCLC	—	—	—	—	POLE p.Gln508Ala509delinsHisSer / RET p.Val53Leu / RET p.Gln70Leu / RET p.Arg417Leu / SMO p.Gly65Cys / TP53 p.Arg158Leu	—	—	—
24	NSCLC (adenocarcinoma)	I-A	*EGFR* p.Glu746_Ser752delinsVal afatinib, dacomitinib, erlotinib, erlotinib + ramucirumab, gefitinib, osimertinib	—	NA	*EGFR* p.Glu746_Ser752delinsVal	—	—	—
26	NSCLC (squamous cell carcinoma)	III-A	*PTEN* p.Glu352* everolimus, tensirolimus (other tumours)	—	NCT04774952	ERBB4 p.Thr371Asn / FGFR2 c.377-1G>A / GNAS p.Pro22Thr / PTEN p.Glu352* / ROS1 p.His1210Leu / TP53 p.Asp184Glufs*63	—	—	—
27	High-grade transitional cell carcinoma of the bladder	III-A	*ARID1A* p.Gln2100* berzosertib (other tumour)	—	NCT04953104	ARID1A p.Gln2100* / CDKN1A p.Pro12Hisfs*19 / CDKN2B p.Asp110Asn / JAK3 p.Asp671Asn / PALB2 p.Glu3* / TP53 p.His179Arg	—	—	—
		III-A	*PALB2* p.Glu3* olaparib (other tumour)	—	NCT04171700				
28	Colon adenocarcinoma	IV-A / NA (resistance)	*KRAS* p.Gly12Val cobimetinib, binimetinib, trametinib / anti-EGFR antibody (resistance)	—	NCT04117087, NCT03948763	KRAS p.Gly12Val / TP53 p.Lys292Glyfs*52	*PTPN1*1 p.Val428Met	*KRAS* p.Gly12Val PROGNOSIS	—
29	NSCLC (adenocarcinoma)	III-A	*NF1* c.6641+1G>C selumetinib, trametinib (other tumour)	—	NCT02465060	BRAF p.Asn581Ile / BRCA2 p.His2537Leu / ERBB4 p.Gly727Ala / FGFR4 p.His727Gln / NF1 c.6641+1G>C / NF1 p.Leu1095Phe / POLE c.3583-2A>T / TP53 c.375+1G>T	*EGFR* p.Gln276His	—	—
		III-A	*POLE* c.3583-2A>T pembrolizumab, nivolumab (other tumours)	—	NCT02715284				
30	NSCLC (poorly differentiated carcinoma)	IV-A	*KRAS* p.Gly12Asp cobimetinib, binimetinib, trametinib	—	NCT03170206, NCT04735068, NCT03948763	GNAS p.Asp466Glu / KRAS p.Gly12Asp / MPL p.Leu234Val / PRKCH p.Asp232Gly / PTEN p.Asp107Val / TP53 p.Arg249Ser / TSC2 p.Val586Leu	—	KRAS p.Gly12Asp PROGNOSIS	—
31	NSCLC (squamous cell carcinoma)	IV-A	*KRAS* p.Gly12Asp cobimetinib, binimetinib, trametinib	—	NCT03170206, NCT04735068, NCT03948763	ERBB4 p.Gly1272Leu / FANCA p.Arg751Trp / GATA3 p.Arg312Glyfs*44 / KDR p.Ile724Met / KRAS p.Gly12Asp / MAP2K2 p.Val283Glu / MET p.Leu920Pro / PDGFRA p.Val1084Met / PIK3CB p.Ile428Phe / TP53 p.Arg249Trp	—	KRAS p.Gly12Asp PROGNOSIS	—
32	NSCLC (adenocarcinoma)	IV-A	*KRAS* p.Gly12Val cobimetinib, binimetinib, trametinib		NCT03170206, NCT04735068, NCT03948763	ATR p.Asn973Ser / KRAS p.Gly12Val / MTOR p.Asp2102Val / PTCH1 p.Arg606Met / RAD51C p.Leu61Val / RICTOR p.Arg1670Trp / RICTOR p.Pro1397Ser / TP53 p.Gln317*	*KRAS* p.Gly12Val	KRAS p.Gly12Val PROGNOSIS	
33	NSCLC (adenocarcinoma)	III-A	*PIK3CA* p.Glu545Lys alpelisib + fulvestrant (other tumour)	—	NCT04073680, NCT0477495	ERBB4 p.Arg103His / KDR p.Gly95Arg / MITF (NM_000248) p.Arg197_Ala198del / PIK3CA p.Glu545Lys / PTCH1 p.Val1137Leu / TP53 p.Leu32Valfs*10	*MET* 1392+1G>A	—	—
34	Pharyngeal squamous cell carcinoma	—	—	—	—	ATR p.Ser1843Phe / TP53 p.Pro190Leu / TP53 p.Glu294*	—	—	—
35	Colon adenocarcinoma	III-A	*NF1* p.Leu1246Cysfs*20 selumetinib, trametinib (other tumour)	—	NCT02465060	FLT3 p.Gln338* / KRAS p.Ala146Val / NF1 p.Leu1246Cysfs*20 / TP53 p.Arg175His	—	—	—
		IV-A / NA (resistance)	*KRAS* p.Gly12Val cobimetinib, binimetinib, trametinib / anti-EGFR antibody (resistance)	—	NCT04117087, NCT03948763)				
36	NSCLC (adenocarcinoma)	—	—	—	—	ALK p.Glu657Gln / ARID1A p.Pro526Ser / FLT3 p.Val106Phe / IDH1 p.Gly310Val / IDH2 p.Met293Ile / INPP4B p.Gly146Arg / JAK1 p.Ile928Met / JAK3 p.Asp1025His / MTOR p.Pro574Ser / NF2 p.Glu442Gln / POLE p.Phe695Ile / TP53 p.Arg273Leu	*TP53* p.Arg273Leu	—	—
37	NSCLC (squamous cell carcinoma)	III-A	*PALB2* p.Gly881Valfs*8 olaparib (other tumour)	—	NCT04171700	KIT p.Arg161Met / PALB2 p.Gly881Valfs*8 / PIK3CA p.Glu542Lys / TP53 p.Arg156Profs*15	—	—	—
		III-A	*PIK3CA* p.Glu542Lys alpelisib + fulvestrant (other tumour)	—	NCT04073680, NCT04774952				
38	NSCLC (adenocarcinoma)	—	—	—	—	*MET* p.Gly872Valfs*4	—	—	—
39	Colon adenocarcinoma	—	—	—	—	IGF1R p.Asp654Val / TP53 p.Asn131Lysfs*42	*TP53* p.Arg249Lys	—	—
40	NSCLC (adenocarcinoma)	III-A	*ATM* p.Tyr2019Cys olaparib (other tumour)	—	NCT03742895	ATM p.Tyr2019Cys / KDR p.Ser971Arg / KRAS p.Gly12Val / PIK3R1 p.Arg574Ile / SMO p.Leu426Pro / TP53 p.Gly154Val	—	*KRAS* p.Gly12Val PROGNOSIS	—
		IV-A	*KRAS* p.Gly12Val cobimetinib, binimetinib, trametinib	—	NCT03170206, NCT04735068, NCT03948763				
41	Colon adenocarcinoma	IV-A / NA (resistance)	*KRAS* p.Gly12Asp cobimetinib, binimetinib, trametinib / anti-EGFR antibody (resistance)	—	NCT04117087, NCT03948763)	AR p.Met788Leu / ATM p.Leu546Val / CCND3 p.Glu253Asp / ERBB2 p.Ala1216Asp / ERBB3 p.Arg488Gln / IGF1R p.Gly1330Ser / IGF2 p.Arg128Lys / KDR p.Cys482Arg / KRAS p.Gly12Asp / MAP2K1 p.Gly301Arg / TP53 p.Arg267Trp / TP53 p.Arg248Gln	—	*KRAS* p.Gly12Asp PROGNOSIS	—
42	Colon adenocarcinoma	I-A	*BRAF* p.Val600Glu encorafenib + cetuximab	—	NA	BRAF p.Val600Glu / BRCA2 p.Ala2951Thr / MPL p.Arg592Gln / TP53 p.Ile232del	—	*BRAF* p.Val600Glu PROGNOSIS	—
43	NSCLC (squamous cell carcinoma)	III-A	*NF1* p.Arg262Valfs*19 selumetinib, trametinib (other tumour)	—	NCT02465060	ALK p.Gly939Ser / ARID1A p.Ala1136Ser / ERBB2 p.Ala21Gly / ERBB4 p.Glu547Asp / GNAS p.Arg201Cys / NF1 p.Arg262Valfs*19 / TP53 p.Ser315Leufs*30	ERBB2 p.Ala21Gly / TP53 p.Ser315Leufs*30	—	—
44	Melanoma	I-A	*BRAF* p.Val600Glu vemurafenib, dabrafenib, trametinib, dabrafenib + trametinib, vemurafenib + cobimetinib, encorafenib + binimetinib	—	NA	BRAF p.Val600Glu / BRCA2 p.Arg2861Lys	—	*BRAF* p.Val600Glu PROGNOSIS	—
45	NSCLC (squamous cell carcinoma)	IV-A	*KRAS* p.Gln61Leu cobimetinib, binimetinib, trametinib	—	NCT03170206, NCT04735068	ALK p.Tyr1092* / ERBB4 p.His470Asp / GNAS p.Ser532Alafs*158 / GNAS p.Ala548Pro / KDR p.Tyr418His / KRAS p.Gln61Leu / TP53 p.Gln192*	—	—	—
46	High-grade glioblastoma	III-A	*EGFR* p.Pro596Leu, *EGFR* amplification N>25 afatinib, dacomitinib, erlotinib, erlotinib + ramucirumab, gefitinib, osimertinib (other tumour)	—	NCT04933422, NCT03618667	EGFR p.Pro596Leu / EGFR amplification N>25 / INPP4B p.Ile83Met / MITF p.Arg13* / PTCH1 p.Tyr373* / STAG2 p.Arg604* / TSC1 p.Pro196Arg	—	—	—
		IV-A	*PTCH1* p.Tyr373* vismodegib, sonidegib	—	—				
		IV-A	*STAG2* p.Arg604* olaparib, veliparib, rucaparib	—	—				
48	SCLC	—	—	—	—	TP53 p.Leu145Gln / TSC2 p.Asp1403Glu	*TP53* p.Leu145Gln	—	—
49	NSCLC (adenocarcinoma)	III-A	*POLE* c.1686+1G>A pembrolizumab, nivolumab (other tumours)	—	NCT02715284	KRAS p.Gly12Val / POLE c.1686+1G>A / SH2B3 p.Asp241Asn / STK11 c.374+1A>G / TP53 c.375+1G>A	KRAS p.Gly12Val / TP53 c.375+1G>A	—	—
		III-A	*STK11* c.374+1A>G everolimus (other tumour)	—	NCT04173507				
		IV-A	*KRAS* p.Gly12Val cobimetinib, binimetinib, trametinib		NCT03170206				
50	SCLC	III-A	*KIT* p.Asp816Val, *KIT* p.Asp820Val sorafenib, ponatinib, nilotinib, dasatinib, regorafenib ripretinib, avapritinib		NCT04771520	KIT p.Asp816Val / KIT p.Asp820Val / TP53 p.Cys135Arg / MTOR p.His1819Asn	KIT p.Asp816Val / KIT p.Asp820Val / TP53 p.Cys135Arg / MTOR p.His1819Asn / GNAS p.Arg547His	—	—

Treatment with highest level of evidence is given.

*NA* not applicable, *SCLC* small cell lung cancer, *NSCLC* non-small cell lung cancer.

**Fig. 5:**
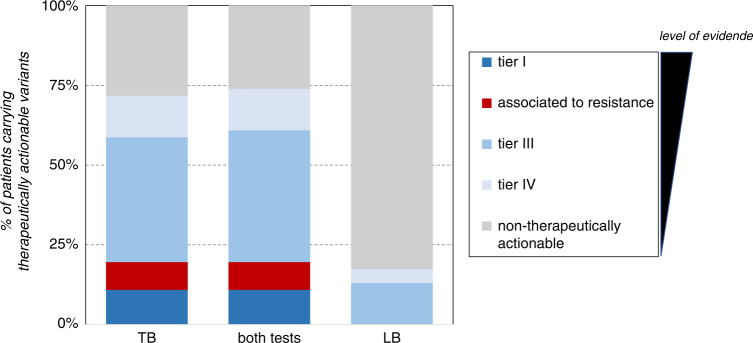
Therapeutical actionability of somatic panels tests. Comparison of findings related to therapeutical actionability between the tissue biopsy (TB) and liquid biopsy (LB) panels. Variants were assessed according to ESMO Scale for Clinical Actionability of molecular Targets (ESCAT) guidelines [62]: 5/46 patients (10.9%) presented a variant ranked tier-I, the highest level of therapeutical evidence, with already approved indications (2 exon 19 *EGFR* deletions in two non-small cell lung cancer (NSCLC) patients, 1 *KRAS* p.Gly12Cys in a NSCLC and 2 *BRAF* p.Val600Glu, in a colon adenocarcinoma and a melanoma), and other 4/46 patients (8.7%) presented a variant associated to resistance to approved therapy (4 *KRAS* p.Gly12Val/p.Gly13Asp in colon adenocarcinomas). All these 9 variants were only detected by TB. 18/46 (39.1%) patients presented at least a variant ranked tier III (hypothetical target for a personalised treatment), opening the possibility of an alternative therapy opportunity if needed. Additionally, 6/46 patients (13.0%), presented a variant ranked tier IV, with preclinical evidence supporting an alteration-drug match. No tier II variants were found. No potential targeted therapies or access to clinical trials was found in 13/46 cases (28.3%). In 27/46 (58.7%) cases potential access to a clinical trial was identified. Conversely, 8/28 ctDNA variants (28.6%), affecting 6 patients, were considered therapeutically actionable. Four patients presented variants ranked tier III and two patients presented a variant ranked tier IV, according to ESMO ESCAT guidelines (Table 3). Therapeutical actionability of both tests together is slightly higher than that reached only by tissue biopsy test (73.9% versus 71.7%) as liquid biopsy revealed the unique therapy associated for one patient.

The tissue biopsy panel identified 51 variants in 33 patients (71.7%) that were considered therapeutically actionable, whereas the liquid biopsy identified 8 variants in 7 patients (15.2%). In 2 of the 46 cases (4.3%), the liquid biopsy panel provided treatment options not identified in the tissue biopsy. However, one of those patients already had higher evidence-level therapy provided by a tissue biopsy panel (Table 3). Variants ranked tier-I (according to ESCAT guidelines [44]) and those associated with resistance to standard therapies, were only found by tissue biopsy panel (Fig. 5). Eleven variants associated with prognosis, detected in 11 patients, were identified by the tissue biopsy panel, while only one was detected by the liquid biopsy panel (Table 3). Additionally, 210 variants identified by either one of both tests could be used as potential personalised ctDNA biomarkers for follow-up. The tissue biopsy panel allowed the identification of potential ctDNA biomarkers for every patient. In contrast, the liquid biopsy did so for only 17 patients (37.0%). Finally, although no clinical suspicion of cancer predisposition was noticed a priori in any of our patients, germline sequencing revealed three pathogenic/likely pathogenic variants associated with familial cancer in three patients (6.5%).

Patient #4, with a squamous cell carcinoma of the lung, was a heterozygous carrier of the likely pathogenic variant *EXT1* c.357 C > A, p.Tyr119*, associated with hereditary multiple osteochondromas [44, 45]. Retrospective revision of previous Positron Emission Tomography and Computed Tomography (PET/CT) images revealed multiple bone lesions in the pelvis compatible with multiple osteochondromas. The patient has a wide family history of cancer, including a “bone cancer” in his deceased mother. Patient #5, a man with pilomatrix carcinoma with lung metastasis, was a heterozygous carrier of the likely pathogenic variant *BRIP1* c.526_536del; p.Phe176Serfs*9. Pathogenic germline variants in *BRIP1* confer a high risk for ovarian cancer [46]. The patient has a compatible family history (early ovarian cancer in paternal aunt and cousin). Patient #10, a woman with uterus carcinosarcoma with lung metastasis without a family history of cancer, was a heterozygous carrier of the pathogenic variant *BRCA1* c.3331_3334del; p.Gln1111Asnfs*5, associated with hereditary breast and ovarian cancer syndrome [47, 48]. None of the three cases fulfilled conventional criteria for germline testing, prompting the classification of these results as clinically useful incidental findings. According to guidelines from the American College of Medical Genetics and Genomics (ACMG), the *BRCA1* variant was considered pathogenic, whereas the *EXT1* and *BRIP1* variants were classified as likely pathogenic (Supplementary Table 4). Regarding clinical actionability, the *EXT1* variant has implications for differential diagnosis [45], the *BRCA1* variant for therapy selection, and both the *BRCA1* and the *BRIP1* variants are clinically relevant for risk assessment, prevention, prophylaxis and early detection of their associated cancers [49] (Supplementary Table 4).

Globally, in our cohort, the IGP approach provides options for improvement in patient’s clinical management in 73.9% (34/46) of cases, facilitating access to personalised therapies, biomarker-guided clinical trials, and/or genetic counselling for the patients and their families.

### Liquid biopsy-based follow-up in radiation oncology

As liquid biopsies can be used to monitor the evolution of tumours over time, we hypothesised that they might provide predictive information before RT, during RT and/or on the follow-up. We focused on two indicators: the total concentration of plasma cfDNA ([cfDNA]) and the presence/absence and AF of tumour-specific ctDNA variants.

Plasma [cfDNA] was quantified in all liquid biopsy samples for all patients. Full [cfDNA] data are displayed in Supplementary Table 5.

ctDNA signal was positive at least at one time point during the RT or follow-up in 33 patients of the 45 tested (73.3%) (ctDNA shedders), and in 31 (68.9%), ctDNA was positive during RT. 62.5% of ES patients (10/16), 64.7% of OMT patients (11/17) and 85.7% of OMT + patients (12/14), were ctDNA shedders. Twelve patients (26.7%) did not have any positive ctDNA signal at any time point using the targeted PCR technique (ctDNA nonshedders). ctDNA dynamics plot for all patients is included in Supplementary Fig. 3.

### Clinical utility of cfDNA and ctDNA analysis before RT

Baseline [cfDNA] (before starting RT) was available from 48 cases (Supplementary Table 5). Plasma [cfDNA] was significantly higher at baseline in patients (10.9 ± 7.2 ng/mL) than in healthy controls (4.4 ± 1.5 ng/mL) (*p*-value = 0.0035, Mann–Whitney U test) (Supplementary Table 6). However, no correlation was found in our cohort between baseline [cfDNA] and stage (*p*-value > 0.05, Mann–Whitney U test), tumour volume or metabolic activity measured as SUVmax in 18F-FDG PET/CT (*p*-value > 0.05, Pearson correlation test), as seen in other works [50]. Baseline [cfDNA] did not differ between ES, OMT and OMT + patients (*p*-value > 0.05, Mann–Whitney U test).

With the aim of exploring tumour-specific biomarkers, beyond total cfDNA levels, we assessed the baseline ctDNA status (presence/absence of the selected tumour biomarkers in baseline liquid biopsy), and its potential correlation with clinical status. Baseline ctDNA data were available for 31 ctDNA shedders. 51.6% of them (16/31) were positive at baseline (baseline ctDNA-positive). The baseline ctDNA-positive rate was different between clinical groups: 30.0% of ES patients (3/10), 50.0% of OMT patients (5/10) and 72.7% of OMT + patients (8/11). Baseline ctDNA-positive patients tended to have larger lesions than the negative ones, although these differences did not reach statistical significance (*p*-value > 0.05, Mann–Whitney U test). There was no difference in baseline [cfDNA] between baseline ctDNA-positive and negative patients (*p*-value > 0.05, Mann–Whitney U test).

**Fig. 6:**
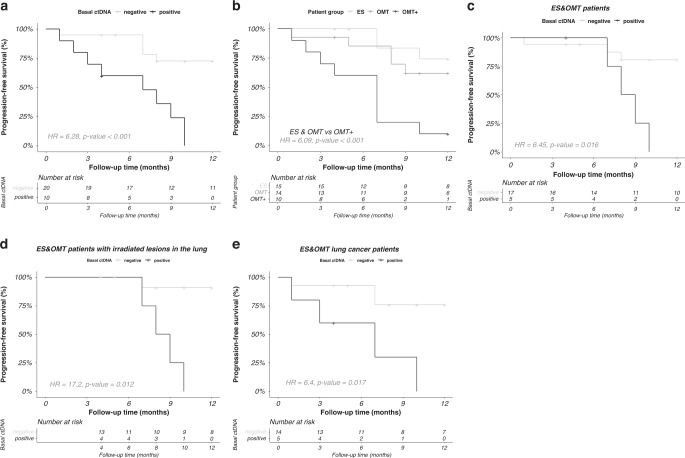
Kaplan–Meier plots of progression-free survival (PFS) after RT treatment. **a** PFS of all patients according to basal ctDNA signal, those not tested are not represented; **b** PFS of all patients according to clinical group; **c** PFS of ES and OMT patients considered together and stratified according to basal ctDNA signal; **d** PFS of ES and OMT patients with irradiated lesions in the lung; **e** PFS of ES and OMT lung cancer patients. Statistical significance (*p*-value) and hazard ratio (HR) were calculated using Cox proportional hazards regression model the in all cases. Below each plot “number at risk” table is shown. Censored subjects are indicated on the Kaplan–Meier curve as tick marks.

Interestingly, baseline ctDNA status correlated with progression-free survival (PFS) after RT. As shown in Fig. 6, PFS was higher in baseline ctDNA-negative patients than in positive ones (Fig. 6a). OMT + patients are mostly ctDNA-positive at baseline and, as expected, progressed significantly earlier after RT than ES and OMT patients (Fig. 6b). It is noteworthy that, restricting the analysis to ES and OMT patients, ctDNA status at baseline also worked as a prognostic marker, even considering only those ES and OMT patients who underwent RT for lesions located in the lung (Fig. 6c, d). This was also true for the subset of ES and OMT patients with lung primaries (Fig. 6e). These observations suggest that basal ctDNA status could be used for the personalisation of treatment and follow-up, potentially providing better prognostic/predictive indicators than clinical status.

### Clinical utility of cfDNA and ctDNA analysis during RT

Considering [cfDNA] dynamics during the treatment, no general trend in response to radiation was observed in our cohort, not even in patients with multiple liquid biopsies after the same RT sessions (Supplementary Fig. 4 and Supplementary Table 5).

The study of ctDNA signal during RT did not reveal common patterns among patients either. In patients with several biomarkers monitored, some of them followed different AF patterns at certain points (e.g. patients #32, #35 and #36, among others) (Supplementary Fig. 3), which could be a reflection of the spatial genetic heterogeneity within the irradiated lesions.

### Clinical utility of cfDNA and ctDNA analysis during the follow-up

Total [cfDNA] seems to be independent of disease status during the follow-up of our cohort (Supplementary Table 5). On the contrary, ctDNA provided predictive information in a significant number of patients. Of the 45 patients in whom the longitudinal ctDNA study was carried out, complete and conclusive clinical follow-up information was obtained for 36 (80.0%). Of those, in 22 (22/36, 61.1%), clinical status and ctDNA signal were concordant (12 cases of disease progression and 10 cases of response to treatment). On the other hand, no correlation was found for 14 patients (14/36, 38.9%), consisting of 11 non-ctDNA shedders during the treatment and 3 patients in which ctDNA remained undetectable during the follow-up in spite of disease progression noted by imaging tests (Supplementary Fig. 3, Supplementary Table 7). Interestingly, for five patients, the ctDNA signal anticipated the observation of relapse on imaging tests (patients #23, #28, #33, #34 and #48; Supplementary Fig. 3). Of these, in two lung cancer cases, liquid biopsy results were suggestive of progression while imaging tests remained inconclusive for months (patients #23 and #33; Supplementary Fig. 3), likely as a consequence of post-RT tissue changes [51].

## Discussion

Precision Medicine requires the integration of molecular and clinical information from different sources in an n-of-one context, presenting interpretation challenges that demand time and expertise. In this work, we interrogate the power of IGP and liquid biopsies to enable Precision Medicine in Radiation Oncology, one of the main therapeutic approaches for the management of cancer patients, but still with many unknowns at the molecular level. We explore a cohort of early-stage and oligometastatic, potentially curable, patients, in whom the whole armamentarium of genomics has a longer time window for its clinical applicability. Our results show that in cancer patients treated with RT, the use of liquid biopsies in the context of IGP goes beyond targeted therapies: it can contribute to adapt standard-of-care treatment, improve follow-up, prevent second primaries, early diagnosis of unknown synchronic tumours and familial genetic counselling.

Regarding the adaptation of standard-of-care treatments, although most ES and OMT patients have a good prognosis a priori, there are currently no individual predictors of patient’s response. Contributing towards personalised RT treatments, ctDNA biomarkers can help in the identification of patients with a high risk of relapse, who could take advantage of RT intensification or other adjuvant treatments. In this study, baseline ctDNA exhibited solid prognostic significance, showing a strong correlation to PFS. To our knowledge, this is the first time such a correlation is shown specifically in early-stage RT patients. Our results in ES and OMT patients are in accordance with those reported during the preparation of this manuscript in patients with stage I-A–III-B NSCLC treated with curative intent with different therapeutic approaches (surgery, chemotherapy and/or RT). In these patients, ctDNA detection before treatment was associated with shorter overall survival and recurrence-free survival [52].

Despite belonging to different clinical stages and histologies, our ES and OMT patients behaved very similarly in terms of PFS. As, to some extent, oligometastatic tumours resemble localised diseases, curative RT techniques are now integrated into oligometastasis management [53]. Our results support this strategy and outline how the same ctDNA-based monitoring approach could be applied to both groups, reinforcing the use of basal ctDNA information to stratify RT patients.

The identification of personalised ctDNA biomarkers was possible thanks to genomic data integration, which enhanced the impact of the information provided by each individual test. Moreover, liquid biopsy confirmed the presence of previous tumour biopsy variants in the active lesions, when tissue biopsies were not available, and germline information enabled the differentiation of true somatic variants either in the tissue biopsy but also in the liquid biopsy. IGP also allowed the discernment of variants potentially derived from CH, which are highly frequent in cancer patients and older individuals and may confound the results [40].

Imaging tests are the gold standard for the follow-up of RT, but, often, necrosis, inflammation or other post-RT tissue changes limit the resolution of these techniques. In this work, liquid biopsy has allowed an optimal molecular follow-up in a significant number of patients, showing a good correlation with established routine surveillance practices in both relapsed patients and good responders. Our data confirm that liquid biopsy stands as a very good complement for those cases of inconclusive imaging results, being capable of detecting molecular disease recurrence earlier than radiologic tests, which would positively affect the outcome. Hence, a liquid biopsy would help to reduce costs and prevent unnecessary medical testing [51].

Since these are, in general, curative-intent treatments, usually no IGP strategies are routinely performed. However, a considerable number of patients relapse. Unfortunately, when genomic testing is the last resource, results usually arrive late, and the patients are no longer eligible for clinical trials. In this study, somatic testing identified alterations associated with approved or investigational therapies in a significant number of patients, serving as a gateway to ongoing clinical trials. The availability of this knowledge prior to the urgency of a new therapy could determine patient survival. On the other hand, germline testing revealed a hereditary predisposition to cancer in 6.5% of patients, modifying their subsequent clinical management according to current clinical guidelines [49].

This work contributes to disclose the utility of liquid biopsy not only in RT but also in the quite still unknown field of low tumour burden patients. The discordances between liquid and tissue biopsy results can be attributed to diverse factors: (1) temporal distance between biopsies: liquid biopsies actually represent the current disease, when comparing temporally matched biopsies, the concordance rate increases; (2) tumour spatial heterogeneity: most of the tissue samples tested came from local biopsies and may not represent the whole tumour or predominant clones and, in the case of oligometastasis, multiple synchronic lesions are present; (3) low ctDNA shedding: especially in earlier stage patients or small lesions; (4) variability of blood ctDNA signal along time: observed AFs are sometimes close to the detection limit, and it is likely that at some point an increase in total cfDNA release will eclipse the ctDNA signal, making it undetectable. Also of note is that the sensitivity to detect CNVs is limited in liquid biopsies. The liquid biopsy panel used in this work only reports gains of 8 or more copies, and its ability to detect them decreases with lower ctDNA fractions, which likely contributed to the lack of CNVs in the results [24].

This work also addresses the actionability of tissue biopsy versus liquid biopsy in low tumour burden patients. In our cohort, the tissue biopsy-based panel achieved better results in terms of therapeutic actionability, identifying alterations associated with therapies in 34 patients, compared to 6 patients with a liquid biopsy panel. This can be explained by the lack of ctDNA variants found by the liquid biopsy test in more than half of the patients. However, the proportion of total detected variants that are therapeutically actionable is slightly higher for the liquid biopsy panel (28.6% versus 23.6%). Likewise, the tissue biopsy panel identified variants that serve as follow-up biomarkers in all patients, whereas the liquid biopsy panel only did so in one-third. Supporting the idea that liquid biopsy gives real-time information about the disease, 88.0% of the variants identified by the liquid biopsy panel that were used as a ctDNA biomarker were detected by the targeted PCR + NGS method in the longitudinal ctDNA study, compared to 67.4% of the variants identified in the tissue biopsy. Even so, all the data above supports the use of liquid biopsy when no tissue biopsy is available in early-stage and oligometastatic patients. Although our study required tissue samples for patient enrolment, it is not always available for HFRT patients, usually affected by non-resectable cancers, a context in which tissue biopsies are likely to fail.

To the best of our knowledge, this is the first study addressing the real-time monitoring of ctDNA release throughout the entire RT treatment and follow-up, and no prior study followed a comparable comprehensive approach. The integration of the different genomic tests is a particular strength of this work; thanks to this approach, biomarker selection is endorsed by well-curated and updated genomic information from each patient.

One of the main milestones for the implementation of ctDNA monitoring in clinical decision-making is the definition of the optimal sampling strategy; our results are limited by the time points analysed during RT, and some transient ctDNA shifts can be missed. More exhaustive research studies are highly warranted to boost progress in real-time therapy adaptation (intensification, de-escalation, interruption, etc.). Another limitation was the logistical difficulties during the post-treatment follow-up; most patients went back to their referring centres, making it impossible to perfectly synchronise liquid biopsies and diagnostic imaging. This also explains most lost to follow-up cases. Additionally, although our in-house method for longitudinal ctDNA analysis is an easy, quick and versatile approach, it is a semi-quantitative technique relying on a large number of PCR cycles with no internal normalisation of AF. The incorporation of unique molecular identifiers (UMIs) in the PCR-based enrichment techniques for integrated error suppression could allow for obtaining more reliable and sensitive results [54, 55]. The use of tailored ctDNA assays informed by prior NGS panels makes the longitudinal analysis more reliable and less susceptible to false positive results, but doesn’t permit the discovery of newly acquired variants during the follow-up. To address this constraint, comprehensive liquid biopsy panels could be periodically inserted between targeted PCR assays, although at the expense of a considerably increased cost. Our work has not addressed the presence of genomic variants that have been proposed to confer radioresistance or radiosensitivity to the tissues treated with RT. Although not firmly established yet, we believe this is an interesting topic, potentially relevant for the personalisation and modulation of RT [56, 57].

In conclusion, IGP provides global and actionable information for personalised decision-making in Radiation Oncology. More specifically, ctDNA characterisation has proven feasible and useful for the clinical management of patients treated with RT. Finally, further efforts are warranted for the development and refinement of ctDNA analysis techniques to reach more sensitive and reliable results in all the scenarios and stages of cancer.

## Materials and methods

### Patients

This study comprised a set of 50 patients (Table 1) who underwent RT in our institution between July 2017 and August 2018, using the Varian TrueBeam STx powered by Novalis linear accelerator (Palo Alto, CA, USA). All patients fulfilled the following criteria: (1) unresectable early-stage or oligometastatic disease (non-surgical candidates), (2) availability of tumour specimen (tissue biopsy or surgical piece) from the lesion to be irradiated and/or its corresponding primary tumour/previous metastasis, (3) life expectancy greater than 3 months, and (4) written consent for participation in the study. Patients’ main medical records were collected before, during and after the treatment, and their response to treatment was assessed according to RECIST 1.1 guidelines: complete response (CR), partial response (PR), stable disease (SD) and progressive disease (PD) [58].

### Radiotherapy

Treatment plans were generated using the Eclipse treatment planning system (Varian Medical Systems, Palo Alto, CA, USA). Planning and delivery were conducted using image-guided volumetric modulated arc therapy (IG-VMAT) with a Varian Truebeam STx Powered by Novalis linear accelerator (Varian Medical Systems, Palo Alto, CA, USA). Coplanar or multiple non-coplanar arcs were used depending on treatment needs. 6MV photon beams were used. Prescription doses to the planning target volume (PTV) and dose constraints to organs at risk were prescribed according to institutional guidelines.

Patient immobilisation with whole body alfa cradle or radiosurgical masks in the central nervous system and head and neck treatments were used to provide accuracy. Patients were CT or PET/CT simulated. Four-dimensional CT (4DCT) was obtained at the time of CT simulation, depending on the area to be treated. Magnetic resonance imaging (MRI) simulation was performed in indicated cases. One millimetre slice thickness reconstructions in the axial plane were transferred to the treatment planning station.

Before each fraction, Exactrac X-Ray 6D image-guided radiotherapy system (Brainlab, Munich, Germany) and kilo-voltage cone-beam CT system was used for patient setup correction.

5/50 patients, with high-grade glioma [1], locally advanced lung cancer [2] and head and neck cancer [2], were treated with CFRT. The gross tumour volume (GTV) received a total dose of 70 Gy in 33–35 fractions, whereas those areas at risk for microscopic spread (clinical target volume, CTV), received a total dose of 60 Gy in 30 fractions. The remaining 45/50 patients were treated with different regimes of HFRT, distributed as follows.

5/50 patients (those with brain metastasis) received radiosurgery (RS) or hypofractionated radiosurgery (HRS), which are non-surgical radiotherapy techniques used to deliver precisely-targeted radiation in a few high-dose fractions. Prescription doses were 15–40 Gy in 1–10 fractions, based on the GTV. PTV was generated by the geometric 2 mm expansion of the GTV.

34/50 patients, with lung nodules, and 3/50 patients, with liver metastasis, underwent SBRT (a technique analogous to RS/HRS but on body regions different from the brain), with prescription doses of 40–60 Gy in 3–8 fractions, depending on GTV and location. Two patients with recurrent lung lesions or prior RT were included. Diagnostic PET scan images and metabolic tumour volume (MTV) were routinely used. Internal tumour volume (ITV) was contoured from 4DCT. PTV was defined by adding 5 mm in all directions from ITV.

3/50 patients, with soft tissue metastasis, 2 in the neck and 1 in the navel, were treated with SBRT and prescription doses of 27–30 Gy in 3–5 fractions.

To compare the effects of the various treatment protocols with different treatment fraction sizes and doses, the biologically effective dose (BED) was calculated using the linear quadratic model: BED = D(1 + d/α/β) where D is the total dose, d is the dose per fraction, and α/β ration for the tumour was 10 Gy.

### Integrative genomic profiling: NGS panels

IGP strategy and study design are schematized in Fig. 1. Tumour tissue and germline IGP were conducted using ONCOgenics Complete^TM^ (IMOMA, Oviedo, Spain), a hybrid capture-based pan-cancer panel for NGS described by Cabanillas et al. [59]. Tumour specimens consisted of formalin-fixed, paraffin embedded (FFPE) from a tissue biopsy/surgical piece in 87.0% (40/46) of the cases and fine needle aspiration (FNA) samples in 13.0% (6/46). In 58.7% (27/46) of the patients, the tissue sample corresponded to the lesion under treatment (temporally matched tissue/liquid biopsies), whereas in 41.3% (19/46), it came from another related lesion: primary tumour [16], previous metastasis [2] or recurrence [1]. Germline DNA was obtained from peripheral blood cells. A liquid biopsy panel test was performed using Foundation ACT™ (Foundation Medicine, Cambridge, MA, USA), from peripheral blood samples collected during RT treatment, mainly drawn after the first RT session for HFRT treatments and at other time points for CFRT treatments (Table 2). This test reports SNVs, indels, selected rearrangements and CNVs (gains with copy number ≥8) on a series of genes and selected gene regions [24]. Automated germline subtraction was integrated in the pipeline of the tissue biopsy subpanel of ONCOgenics Complete^TM^ in order to define the somatic status of the variants; synonymous and intronic variants, as well as CNVs with copy numbers below 8 were filtered out. Germline-subpanel results were also used to unequivocally identify germline and CH-derived variants in liquid biopsy panels. Briefly, we considered that all variants present in the germline sample with variant allele frequencies equal or above 20% were actually germline and, so, those present in the liquid biopsy list were labelled as “germline” in Supplementary Table 2. Regarding variants present in the germline sample with variant allele frequencies below 20%, we reviewed them manually and experimentally checked whether they were clonal hematopoiesis variants by analysing two independent germline samples per patient by the targeted PCR and deep NGS technique. Those variants consistently found in both independent germline samples with allele frequencies compatible with clonal hematopoiesis were labelled as “CH”.

For the analytical comparison of obtained genomic results between the tissue biopsy and liquid biopsy analyses, only genomic regions covered by both platforms were considered. The clinical significance of all germline genetic variants was evaluated according to ACMG guidelines as pathogenic, likely pathogenic, VUS, likely benign or benign [60]. Clinical significance of all tumour genetic variants was classified according to ESMO Scale for Clinical Actionability of molecular Targets (ESCAT) guidelines: tier-I, targets ready for implementation in routine clinical decisions; tier II, investigational targets that likely define a patient population that benefits from a targeted drug but additional data are needed; tier III, a clinical benefit previously demonstrated in other tumour types (III-A) or for similar molecular targets (III-B); tier IV, preclinical evidence of actionability; variants ranked tier X, with lack of actionability evidence, are not included [61]. Clinical evidence was collected from ONCOKB [62] and CIVIC [63] databases, or PubMed. Clinical trials information was collected from ClinicalTrials.gov database (https://clinicaltrials.gov). Databases last accessed 11 August 2021.

The somatic mutational landscape of our cohort was illustrated as different oncoplots which were compared to the mutational landscape from the lung cancer cohort from the public dataset from Clinical Proteomic Tumor Analysis Consortium 3 (CPTAC-3). Data were downloaded from the repository of the National Cancer Institute (NCI)‘s Genomic Data Commons (GDC), accessed on 26 September 2022, from https://portal.gdc.cancer.gov. We used the Bioconductor R package Maftools [64] version 2.12.0 to create the CPTAC-3 oncoplot.

### Serial blood sample collection and cfDNA obtention

Peripheral blood samples were collected in EDTA tubes prior to each RT session (pre-RT), following the session (post-RT, with different timelags, detailed in Supplementary Table 5) and periodically during follow-up (Fig. 1b). Post-RT blood samples were drawn within the first hour after the end of the session: in 72.0% of the cases [36], post-RT samples have a timelag of 30 min; in 16.0% [8], 60 min; and in 8.0% [4], 15 min. Four patients underwent multiple post-RT blood sampling with different timelags (15, 30, 45 and 60 min). Follow-up liquid biopsies were collected periodically after the treatment (after 1 month and then on a quarterly basis), and were not fully synchronised to diagnostic imaging tests. At least 4 months follow-up was performed in 80.0% of the cases (40/50), no follow-up liquid biopsy could be obtained for 4 patients (4/50 = 8.0%, 3 dropouts and 1 decease), and just 1-month follow-up was completed for 6 patients (6/50 = 12.0%, 3 dropouts and 3 deceases). Plasma was isolated from blood samples within 30 min of blood collection by centrifugation at 2000 × *g* during 10 min at 4 °C, followed by a second centrifugation of the supernatant at 16,000 × *g* during 10 min at 4 °C. Plasma was finally aliquoted in 1–2 mL Nalgene® cryotubes (Merck, Kenilworth, NJ, USA) and stored at −80 °C until cfDNA isolation. Plasma samples were thawed at room temperature, and cfDNA was isolated from 1 to 2 mL aliquots of plasma using the MagMAX™ Cell-Free DNA Isolation Kit (Thermo Fisher, Waltham, MA, USA), following the manufacturer’s instructions. cfDNA samples were analysed and quantified by TapeStation High Sensitivity DNA assay (Agilent Technologies, Santa Clara, CA, USA). Serial peripheral blood samples were collected from 8 healthy donors emulating those from the patients before and after a RT session and were processed in the same way.

### Selection of follow-up biomarkers

For each patient, a selection of confirmed tumour variants was chosen as biomarkers for longitudinal monitoring of ctDNA. Variant selection was based on the following criteria, trying to select at least one variant from each of the three groups per patient: (1) variants identified by liquid biopsy panel, (2) variants identified by both (tissue and liquid) panels (3) variants identified by tissue biopsy panel prioritising: higher AF, oncogenic versus VUS clinical consequence, affected region covered by both panels.

At least one biomarker was selected in 45 patients. A total of 109 variants were assessed (ranging 1–5 per patient): 82 (75.2%) oncogenic/likely oncogenic and 27 (24.8%) VUS/likely benign/benign; at least 1 oncogenic/likely oncogenic variant was evaluated in 44/45 patients (97.8%). 76/109 (69.7%) were detectable by both panels, and 33/109 (30.3%) were only detectable by the tissue biopsy panel. Regarding the variants affecting regions covered by both panels: 16/76 (21.1%) were actually detected by both panels, 51/76 (67.1%) only by the tissue biopsy panel and 9/76 (11.8%) only by the liquid biopsy panel.

### Longitudinal monitoring of ctDNA

Selected biomarkers were interrogated in 649 serial cfDNA samples from 45 patients by targeted PCR and deep NGS sequencing techniques. Starting from 1–10 ng of cfDNA, PCR was carried out using oligonucleotides with specific sequences flanking the selected variant plus 3’ tails with non-specific sequences corresponding to the 5’ part of Illumina adapters. The remaining adapters (including the sample-specific indexes) was added by a second PCR. PCR oligonucleotides are described in Supplementary Table 8. PCR reactions were carried out using Herculase II Fusion Enzyme kit following manufacturer’s instructions (Agilent Technologies). PCR products were purified using Agencourt AMPure XP magnetic beads (Beckman Coulter, Pasadena, CA, USA). Purified PCR products were quantified by TapeStation before sequencing in a NextSeq® 550 platform (Illumina, San Diego, CA, USA). Sequencing results were analysed by an in-house bioinformatics algorithm developed in Python and described in Supplementary Methods. The technique was validated with ctDNA-positive controls reaching 93.7% specificity and 95.9%/95.2%/89.9%/79.5% sensitivity for variants with allele frequencies above 0.4%/0.3%/0.2%/0.1%, respectively (Supplementary Methods). In parallel to all the ctDNA samples, two independent germline samples from each patient were also tested for the variants in all the experiments in order to discard false positive results, CH- or germline-origin.

ctDNA dynamics and clinical status were considered concordant when at least one liquid biopsy test was positive during RT treatment and, during the follow-up, ctDNA signal remained negative in responders or positive at disease progression. When liquid biopsies and diagnostic imaging tests were performed more than five months apart (follow-up desynchronization), it was assumed that no conclusion about a correlation can be taken, and the previous time points were considered for concordance evaluation. Liquid biopsy was considered to anticipate relapse when an increase in ctDNA signal is observed compared to previous liquid biopsy in two consecutive time points, at least for 1 biomarker.

### Statistics

Normality was assessed using Kolmogorov–Smirnov test, and equality of variances, using Levene’s test. Differences between two groups were compared by parametric Student’s *t*-test or non-parametric Mann–Whitney U test. Associations between two continuous variables were studied using Pearson’s correlation coefficient. Chi-squared test was used to evaluate the association between two categorical variables. Progression-free survival (PFS) was defined as the survival time from the completion of RT to the disease progression revealed on an imaging test or death from cancer. Patient cases with no progression or death events were censored at the date of the last follow-up. Survival curves were represented using the Kaplan–Meier method, and comparisons were performed using the Cox proportional hazards regression. For statistical analysis, data were analysed using Microsoft Excel and R software version 4.1.2 and RStudio version 2021.9.0.351.

## Supplementary information

Supplementary methods

Supplementary material legends

Supplementary figures 1, 2, 3

Supplementary table 1

Supplementary table 2

Supplementary table 3

Supplementary table 4

Supplementary table 5

Supplementary table 6

## Data Availability

Relevant data presented in the study are included in the article/supplementary material and further inquiries can be directed to the corresponding authors.

## Code availability

The code for analysis of NGS data from the targeted PCR technique is available upon request.
