# Supplementary material for: Clinical utility of liquid biopsy and integrative genomic profiling in early-stage and oligometastatic cancer patients treated with radiotherapy
Source: Br J Cancer. 2022 Dec 22;128(5):857–76. doi: 10.1038/s41416-022-02102-z (PMC9977775; doi:10.1038/s41416-022-02102-z)
Supplement: Supplementary file 1 — Supplementary methods [file 41416_2022_2102_MOESM1_ESM.docx]

**Supplementary Methods**

1. **Design of the gene panels**

**Liquid-biopsy panel (Foundation ACT):**

Entire coding sequence:

BRCA1

BRCA2

CCND1

CD274

CDH1

CDK4

CDK6

CDKN2A

CRKL

EGFR

ERBB2

ERRFI1

FGFR1

FGFR2

FOXL2

KRAS

MDM2

MET

MYC

MYCN

NF1

PDCD1LG2

PTEN

PTPN11

SMO

TP53

VEGFA

Selected Exons:

ABL1

AKT1

ALK

ARAF

BRAF

BTK

CTNNB1

DDR2

ESR1

EZH2

FGFR3

FLT3

GNA11

GNAQ

GNAS

HRAS

IDH1

IDH2

JAK2

JAK3

KIT

MAP2K1

MAP2K2

MPL

MTOR

MYD88

NPM1

NRAS

PDGFRA

PDGFRB

PIK3CA

RAF1

RET

TERT

Selected Rearrangements:

ALK

EGFR

FGFR3

PDGFRA

RET

ROS1

**ONCOgenics Complete**

- **Tissue-biopsy subpanel**

Entire coding sequence:

ABL1

AKT1

ALK

AR

ARID1A

ARAF

ATM

ATR

BAP1

BRAF

BRCA1

BRCA2

BTK

CBL

CCND1

CCND2

CCND3

CD274

CDK4

CDK6

CDKN1A

CDKN1B

CDKN2A

CDKN2B

CDKN2C

CHEK2

CRKL

CSF1R

CSF3R

CTNNB1

DDR2

EGFR

EPHA2

ERBB2

ERBB3

ERBB4

FANCA

FBXW7

FGFR1

FGFR2

FGFR3

FGFR4

FLCN

FLT3

FRS2

GATA3

GNA11

GNAQ

GNAS

HDAC2

HGF

HRAS

IGF1R

IDH1

IDH2

IGF2

IL7R

INPP4B

JAK1

JAK2

JAK3

KDR

KIT

KRAS

MAPK1

MAP2K1

MAP2K2

MET

MITF

MPL

MTOR

MYD88

NF1

NF2

NOTCH1

NRAS

PALB2

PDGFRA

PDPK1

PIK3CA

PIK3CB

PIK3R1

PIK3R2

PLCG2

POLE

PRKCH

PTCH1

PTEN

RAC1

RAD51C

RAF1

RB1

RET

RICTOR

ROS1

SH2B3

SMO

SOCS1

SRC

STAG2

STK11

TP53

TSC1

TSC2

VEGFA

Selected rearrangements:

ALK

BCR

BRAF

ERBB4

FGFR2

FGFR3

JAK2

MET

NRG1

PDGFB

PDGFRA

RAF1

RET

ROS1

TFE3

TMPRSS2

- **Germline subpanel**

Entire coding sequence:

ALK

APC

ATM

AXIN2

BAP1

BARD1

BLM

BMPR1A

BRAF

BRCA1

BRCA2

BRIP1

BUB1B

CBL

CDH1

CDK4

CDKN1B

CDKN1C

CDKN2A

CHEK2

CYLD

DDB2

DICER1

DIS3L2

DKC1

EGFR

EPCAM

ERCC1

ERCC2

ERCC3

ERCC4

ERCC5

EXT1

EXT2

FAH

FANCA

FANCB

FANCC

FANCD2

FANCE

FANCF

FANCG

FANCI

FANCL

FANCM

FAS

FASLG

FH

FLCN

GPC3

GREM1*

H19

HFE

HRAS

ITK

KCNQ1OT1

KHDC3L

KIT

KRAS

LZTR1

MAX

MC1R

MEN1

MET

MLH1

MNX1

MRE11A

MSH2

MSH6

MTAP

MUTYH

NBN

NF1

NF2

NHP2

NLRP7

NOP10

NSD1

NTHL1

PALB2

PDGFRA

PHOX2B

PMS2

POLD1

POLE

POLH

POT1

PRF1

PRKAR1A

PRSS1

PTCH1

PTCH2

PTEN

PTPN11

RAD51C

RAD51D

RAF1

RB1

RECQL4

RET

RHBDF2

RIT1

RPL11

RPL35A

RPL5

RPS19

RPS24

RPS7

RTEL1

RUNX1

SBDS

SDHA

SDHAF2

SDHB

SDHC

SDHD

SEC23B

SERPINA1

SH2D1A

SHOC2

SLC25A13

SLX4

SMAD4

SMARCA4

SMARCB1

SOS1

SOS2

SPINK1

STIM1

STK11

SUFU

TERC

TERT^#^

TINF2

TMEM127

TP53

TRIM37

TSC1

TSC2

UBE2T

UNC13D

VHL

WNT10A

WRAP53

WRN

WT1

XIAP

XPA

XPC

*only duplications, ^#^ only promoter

1. **Targeted PCR and deep sequencing (NGS) - Python algorithm:**

Sequencing of ctDNA multiplex targeted PCR libraries was performed on a NextSeq500 sequencer (Illumina, CA, USA), following the manufacturers specifications.

A Python customized algorithm was developed for data analysis. Quality control of raw FASTQ files was evaluated using FastQC (v0.11.8). The regions of interest do not lie in flanks of reads so trimming was not necessary. The reads were filtered taking a base quality greater or equal than 20 as threshold. It emulates a simplified alignment, SAM/BAM files are not generated, saving time and resources. For single nucleotide variations and small indels, given the genomic coordinates (GRCh37) of the variant to be interrogated, the algorithm creates a window (32 nucleotides) containing the region of interest. The script generates the reference sequence and the sequence of the alteration. Filtered reads are compared against both sequences. If there is more than one single nucleotide variation on each flanking region, the read is discarded. Considering the reads which match the sequence of the alteration and the reference, observed allele frequency (OAF) is calculated. On the other hand, other value is provided by the algorithm: variant marginal frequency (Q), which represents the frequency of the variant of interest compared to all the reads different from reference in the nearby of the variant position. For this calculation, on the positions -2, -1, +1 and +2 any change is allowed. INDELs are complex alterations and our custom script has some limitations. For this reason, they require a second analysis using command line to check the previous results. Only variants showing AF ≥ 0.1% and Q ≥ 25% were considered positive.

1. **Sensitivity and specificity of the targeted PCR + deep NGS technique**

Sensitivity and specificity of the targeted PCR and deep NGS sequencing technique was calculated using known positive controls. Positive control samples consisted in 2 real cfDNA samples characterized with Foundation ACT liquid biopsy panel. Each cfDNA sample carried 4 ctDNA variants and their specific AF were known.

For sensitivity determination: Serial dilutions of characterized samples were performed to design a set of cfDNA samples with known genetic variants of known AF, ranging 5-0.1 %. Multiplexed targeted PCR was performed as described in “Methods” starting from 5/10 ng of cfDNA. Sensitivity was calculated for expected AF ≥ 0.001, AF ≥ 0.002, AF ≥ 0.003, and AF ≥ 0.004, as the proportion of true positive variants (TP) compared to the number of all data analysed (TP and false negatives, FN), applying the established quality thresholds OAF ≥ 0.1 % and Q ≥ 25 %.

Sensitivity (AF ≥ 0.001) = (97 TP) / (122 TP + FN) = 79.5 %

Sensitivity (AF ≥ 0.002) = (89 TP) / (99 TP + FN) = 89.9 %

Sensitivity (AF ≥ 0.003) = (80 TP) / (84 TP + FN) = 95.2 %

Sensitivity (AF ≥ 0.004) = (71 TP) / (74 TP + FN) = 95.9 %

For specificity determination: using the data from the same experiment, specificity was calculated as the proportion of false positive variants (FP) compared all the genomic positions analysed (consisting of the regions amplified in the targeted PCR assays), applying the established quality thresholds OAF ≥ 0.1 % and Q ≥ 25 %.

Specificity = (252 FP) / (3989 positions) = 93.7 %
