# Supplementary material for: Clinical utility of liquid biopsy and integrative genomic profiling in early-stage and oligometastatic cancer patients treated with radiotherapy
Source: Br J Cancer. 2022 Dec 22;128(5):857–76. doi: 10.1038/s41416-022-02102-z (PMC9977775; doi:10.1038/s41416-022-02102-z)
Supplement: Supplementary file 2 — Supplementary material legends [file 41416_2022_2102_MOESM2_ESM.docx]

**Supplementary material Legends**

**SUPPLEMENTARY FIGURES**

**Supplementary Figure 1. Oncoplot displaying the somatic landscape of our full cohort, our lung cancer cohort and lung cancer patients public dataset from Clinical Proteomic Tumour Analysis Consortium 3 (CPTAC-3).**

**Supplementary Figure 2. Concordance between tissue biopsy and liquid biopsy tests in lung cancer patients.**

**Supplementary Figure 3. ctDNA dynamics of all patients and response assessment (according to RECIST criteria)**

**Supplementary Figure 4. On-treatment [cfDNA] dynamics for patients who underwent multiple post-session liquid biopsies.**

**SUPPLEMENTARY TABLES**

**Supplementary Table 1. Extended demographics, clinical characteristics and treatment details of the cohort.**

**Supplementary Table 2. Variants detected by liquid biopsy panel test and its origin.**

**Supplementary Table 3. Somatic variants detected by tissue biopsy panel and liquid biopsy panel tests.**

**Supplementary Table 4. Clinically relevant germline variants.**

**Supplementary Table 5. Serial peripheral blood drawns and [cfDNA] data from the patients.**

**Supplementary Table 6. Serial peripheral blood drawns and [cfDNA] data from the healthy controls.**

**Supplementary Table 7. Clinical status and ctDNA correlation.**

**Supplementary Table 8. Oligonucleotides for multiplex targeted PCR + deep NGS**
