# Supplementary material for: Clinical utility of liquid biopsy and integrative genomic profiling in early-stage and oligometastatic cancer patients treated with radiotherapy
Source: Br J Cancer. 2022 Dec 22;128(5):857–76. doi: 10.1038/s41416-022-02102-z (PMC9977775; doi:10.1038/s41416-022-02102-z)
Supplement: Supplementary file 3 — Supplementary figures 1, 2, 3 [file 41416_2022_2102_MOESM3_ESM.pdf]

Supplementary Figures

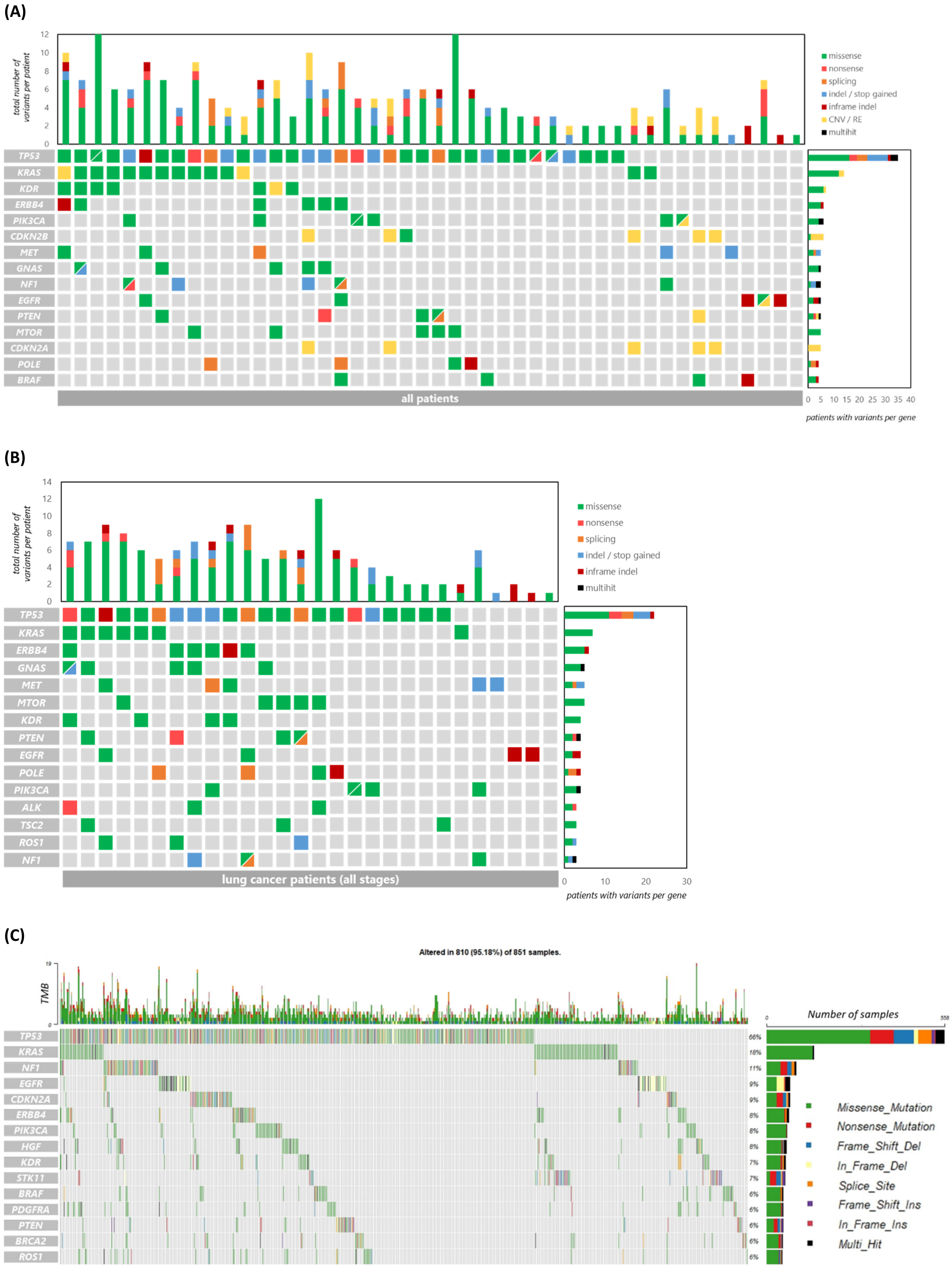

Supplementary Figure 1. *Oncoplot displaying the somatic landscape of our full cohort, our lung cancer cohort and lung cancer patients public dataset from Clinical Proteomic Tumour Analysis Consortium 3 (CPTAC-3).*

For panels (A) and (B), squares with two different sections show multiple hits in the same gene in the patient and are shown in the stacked histogram on the right as "multihit", in black. The stacked histograms on the top of each panel represent the number of variants for each patient considering all the genes in the panel test. Only the 15 most mutated genes are shown in each panel.
(A) *oncoplot of all the patients of our cohort, showing variants detected by tissue and liquid biopsies tests.*
(B) *oncoplot of all the lung cancer patients of our cohort, showing variants detected by tissue and liquid biopsies tests. For the sake of comparison with panel C, CNVs are not represented in this panel.*
(C) *oncoplot of lung cancer patients public dataset from Clinical Proteomic Tumour Analysis Consortium 3 (CPTAC-3). This figure represents the variants detected by tissue biopsy sequencing (after germline sequencing subtraction) of a cohort of 916 cases of lung cancer from the CPTAC-3 project. Only genes included in our tissue and liquid biopsy panels are shown.* Data was downloaded from data repository of the National Cancer Institute (NCI)'s Genomic Data Commons (GDC), from the Cancer Genome Atlas (TCGA) project.
indel, small insertion/deletion; CNV, copy number variation; RE: rearrangement; TMB: tumour mutation burden.

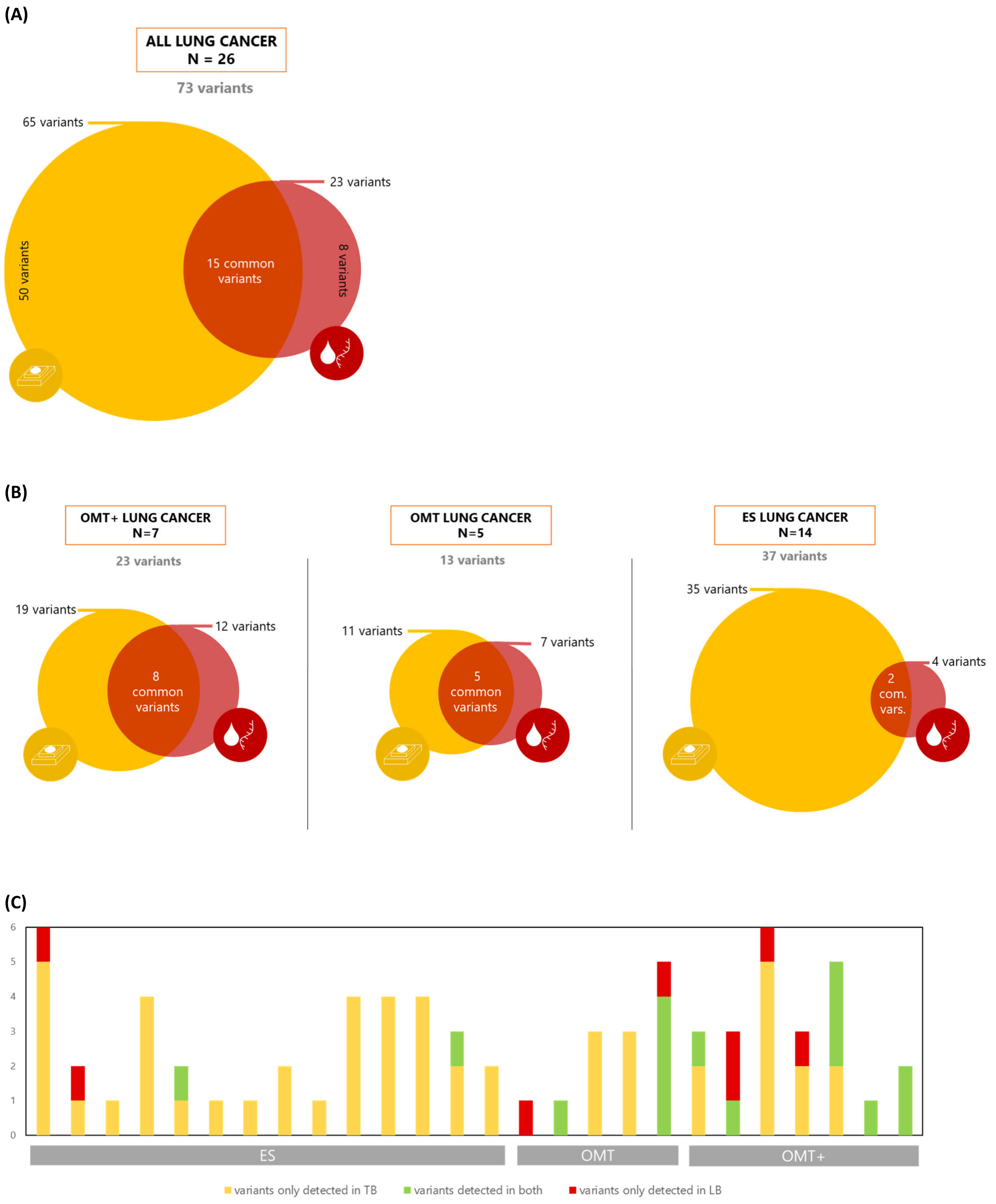

Supplementary Figure 2. Concordance between tissue biopsy and liquid biopsy tests in lung cancer patients.

(A) *Venn diagram for liquid biopsy panel and tissue panel concordances for all lung cancer patients.*

(B) *Venn diagram for liquid biopsy panel and tissue panel concordances plotted separately according to clinical stage group, just considering lung cancer patients.*

(C) *Stacked column chart showing the variant concordance between the panel test for each patient, just considering lung cancer patients.*

In all panels, only genomic regions covered by both platforms are considered. Germline and CH-derived variants are excluded for comparison. Coincident variants are more frequent in advanced-stage patients. TB, tissue biopsy; LB, liquid biopsy.

- **Patient 1**

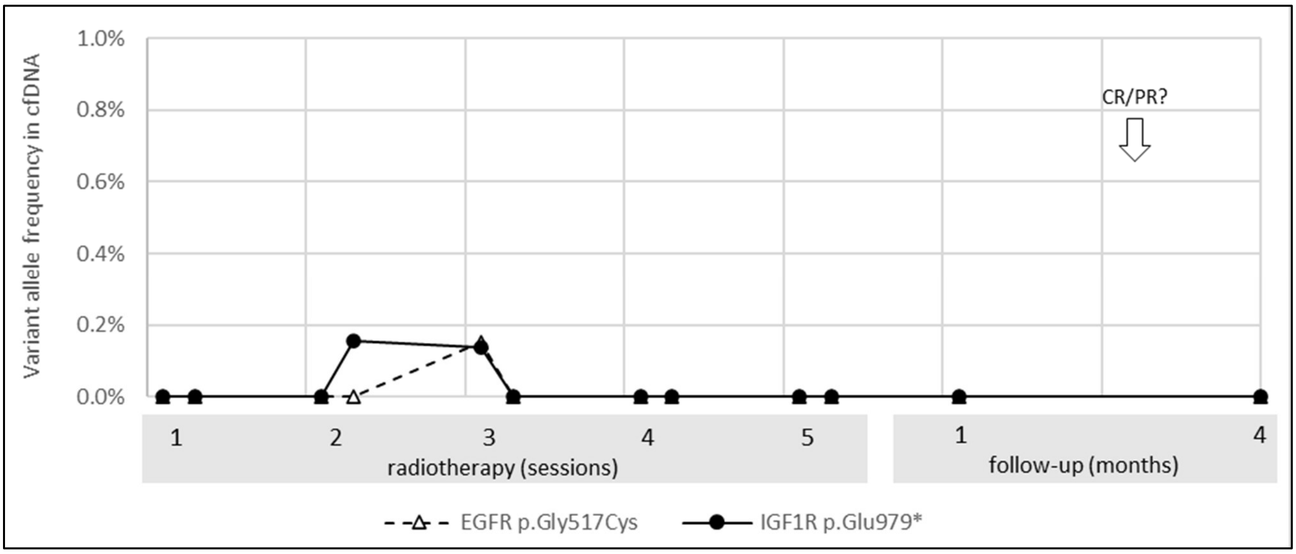

CONCORDANCE

- **Patient 2**

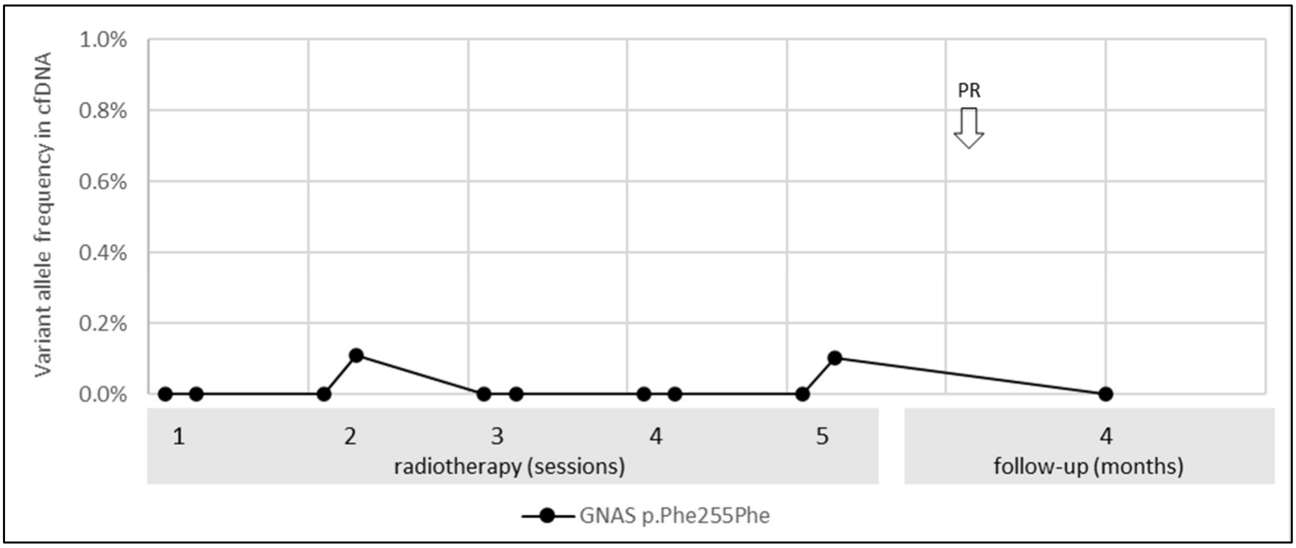

CONCORDANCE

- **Patient 3**

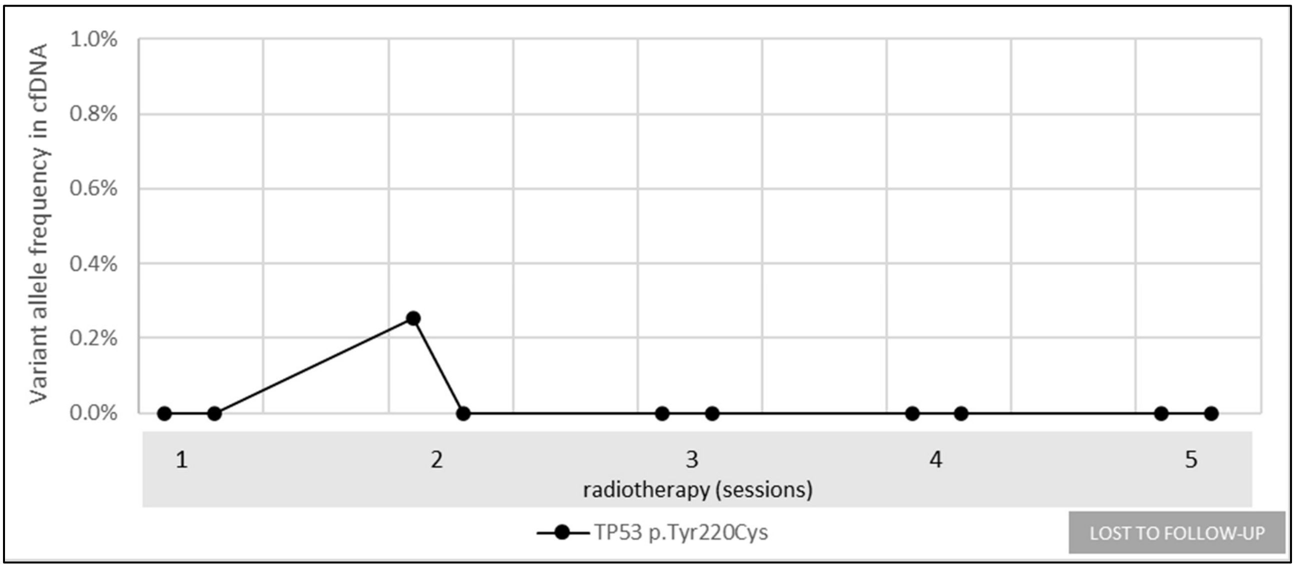

LOST TO FOLLOW-UP

- **Patient 4**

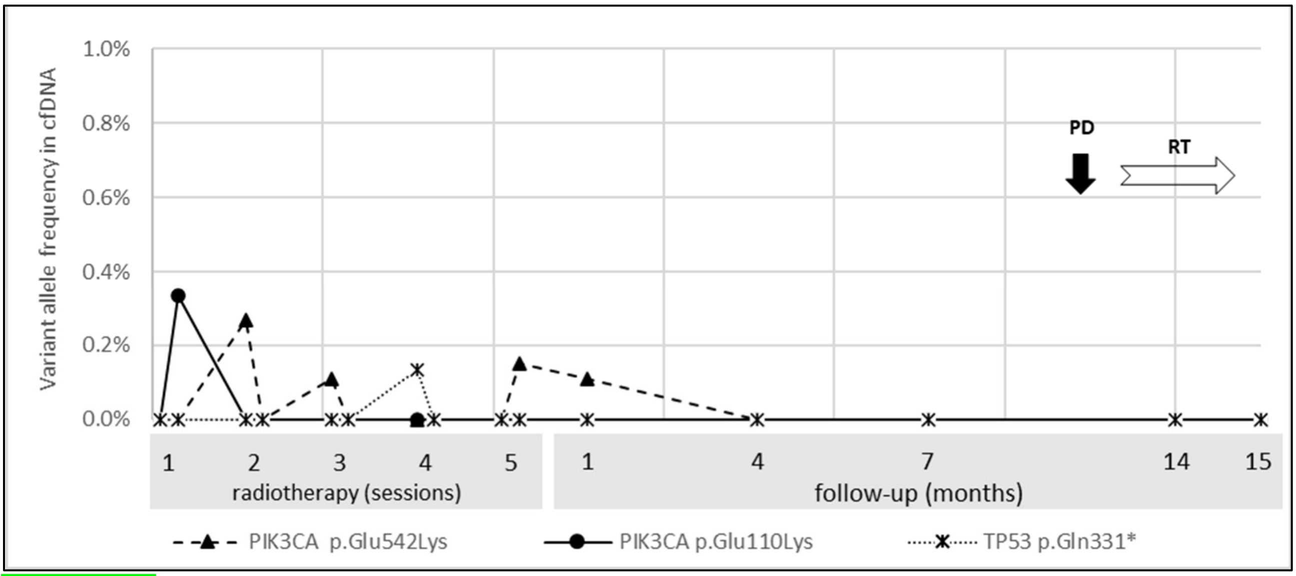

CONCORDANCE

- **Patient 5**

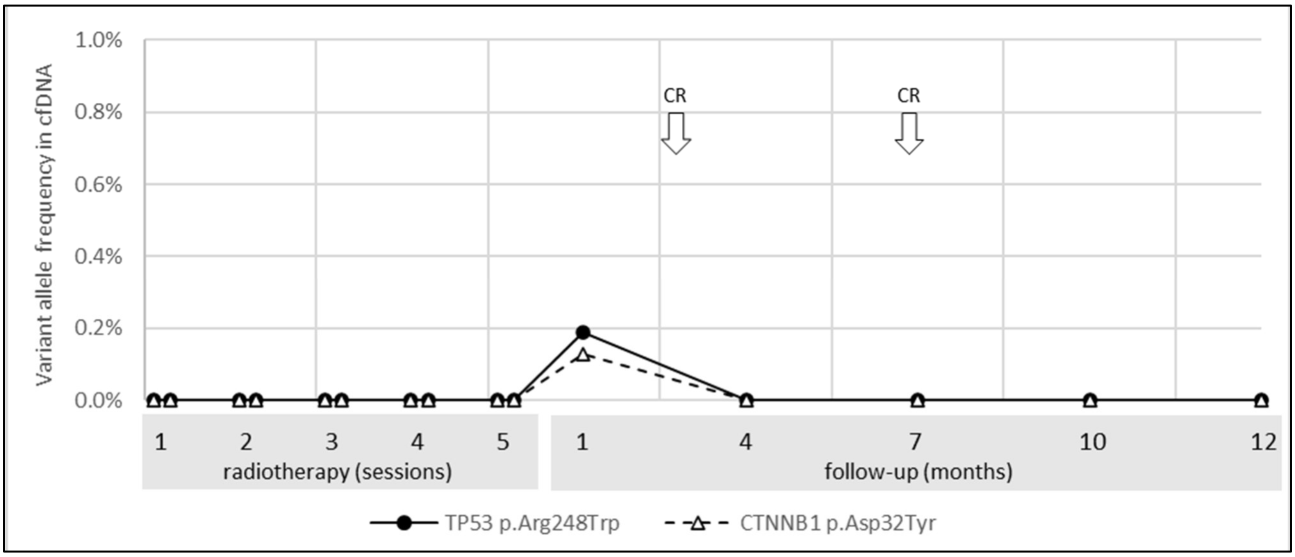

NO CONCORDANCE

- **Patient 6**

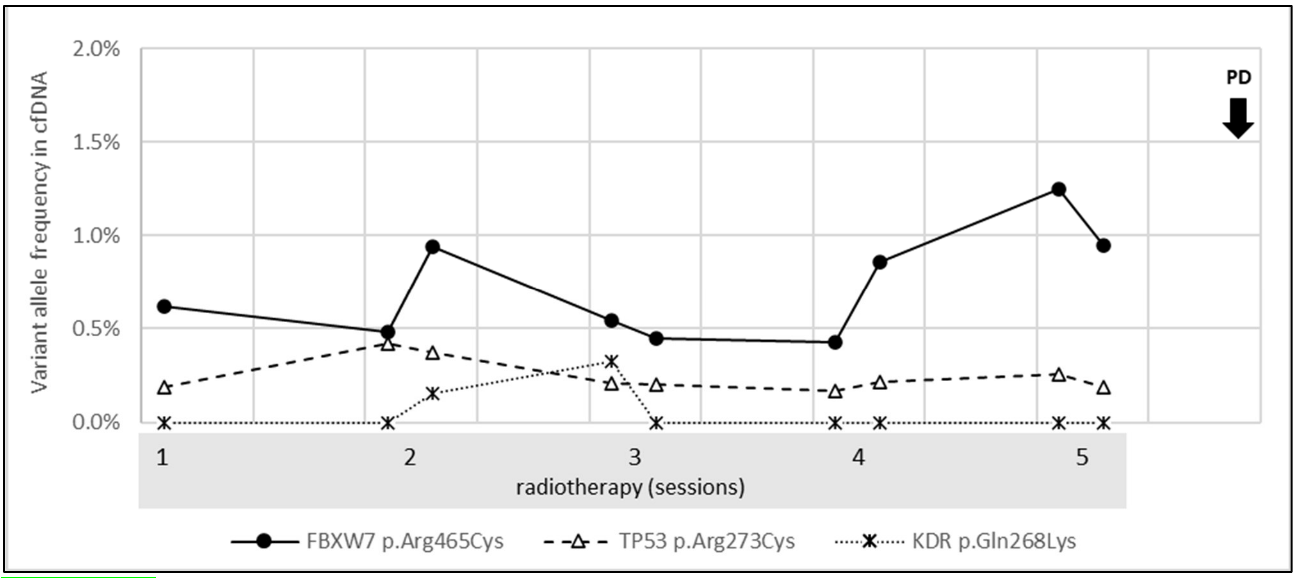

CONCORDANCE

- **Patient 7**

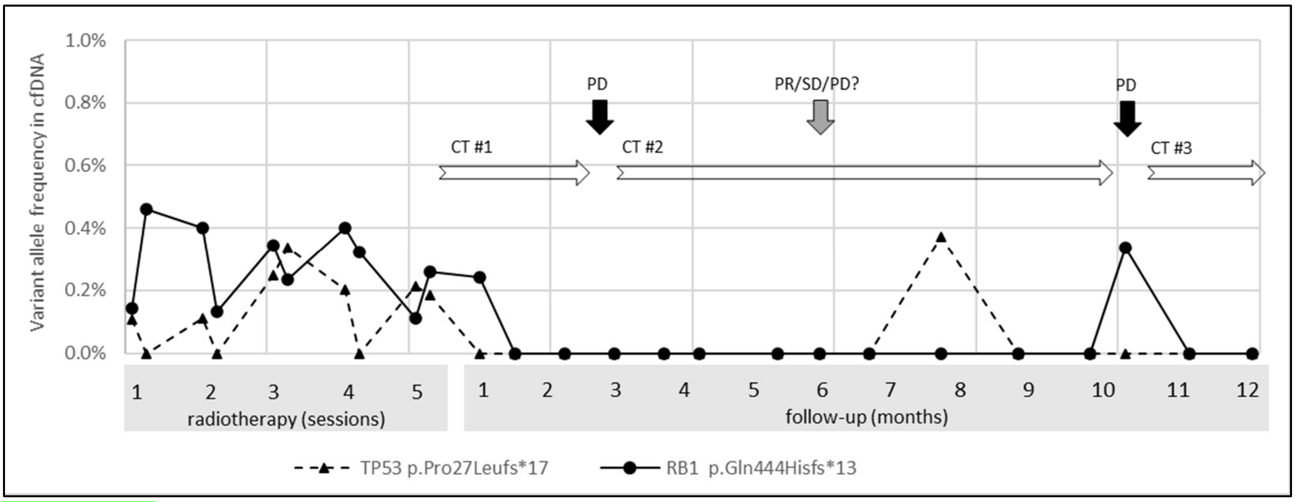

CONCORDANCE (UNTIL CT)

- **Patient 8**

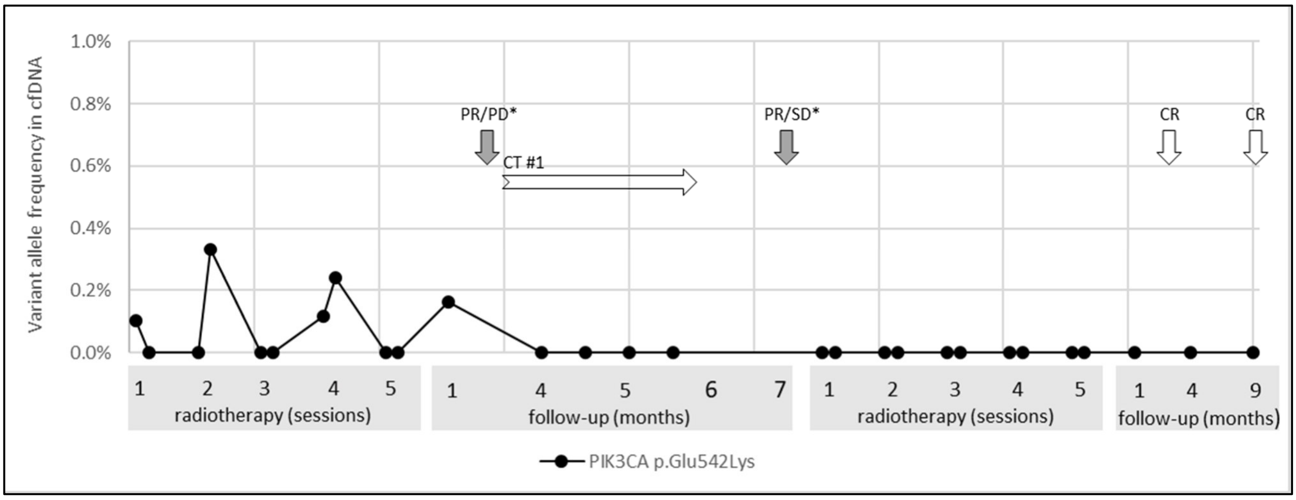

CONCORDANCE (until CT)

- **Patient 14**

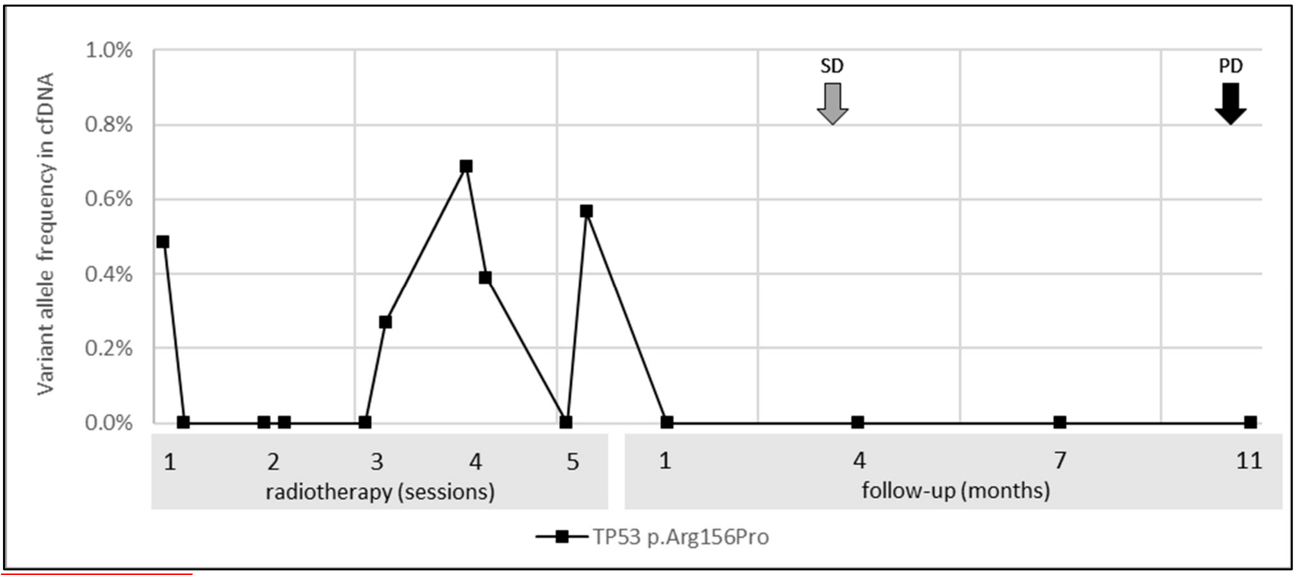

NO CONCORDANCE

- **Patient 16**

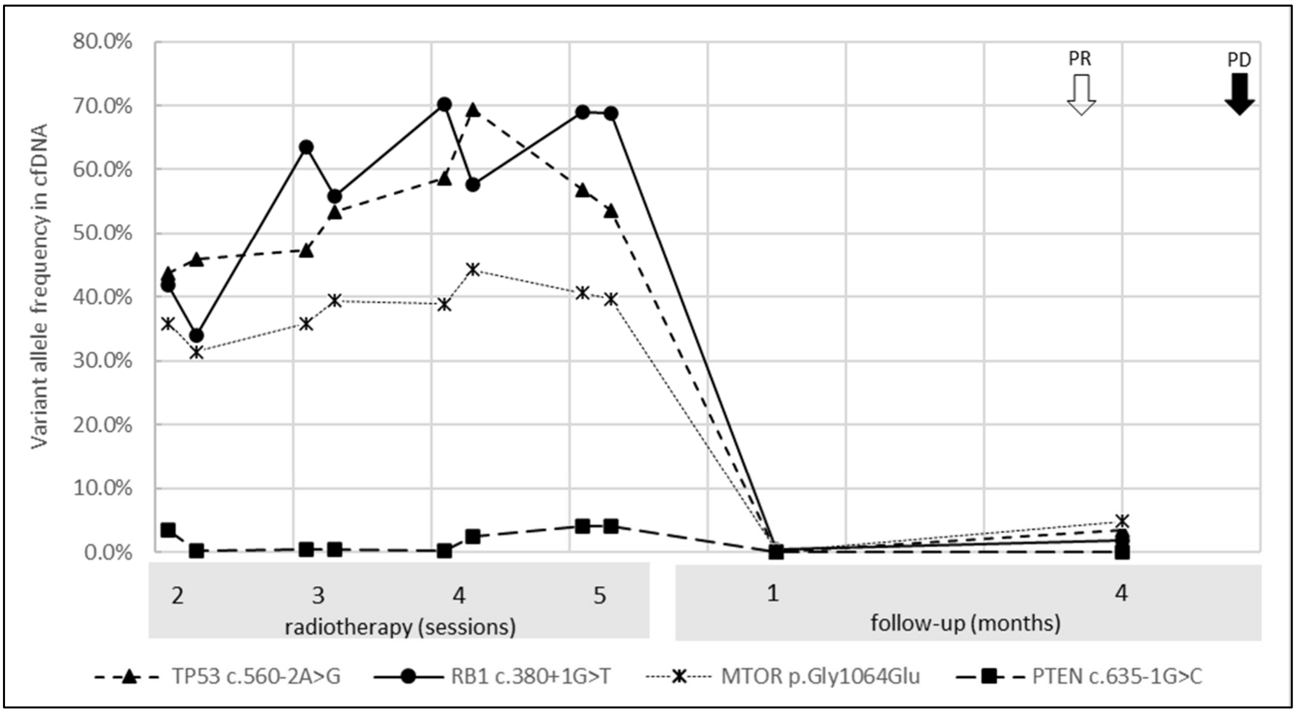

CONCORDANCE

- **Patient 17**

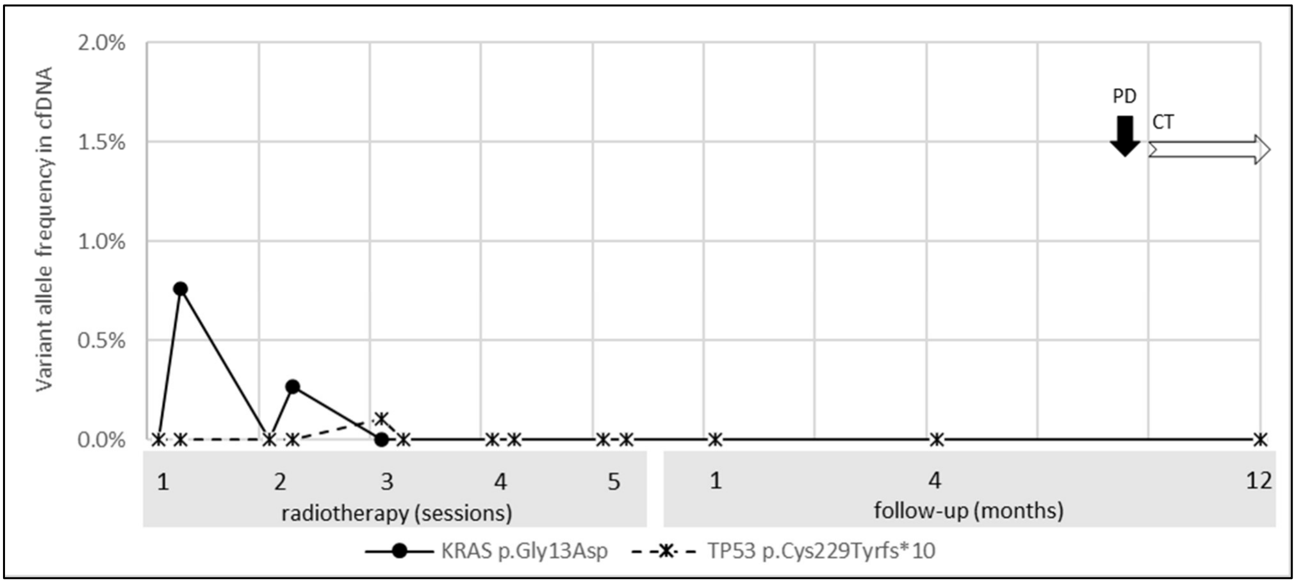

CONCORDANCE (until follow-up desynchronization after 4 months liquid biopsy)

- **Patient 18**

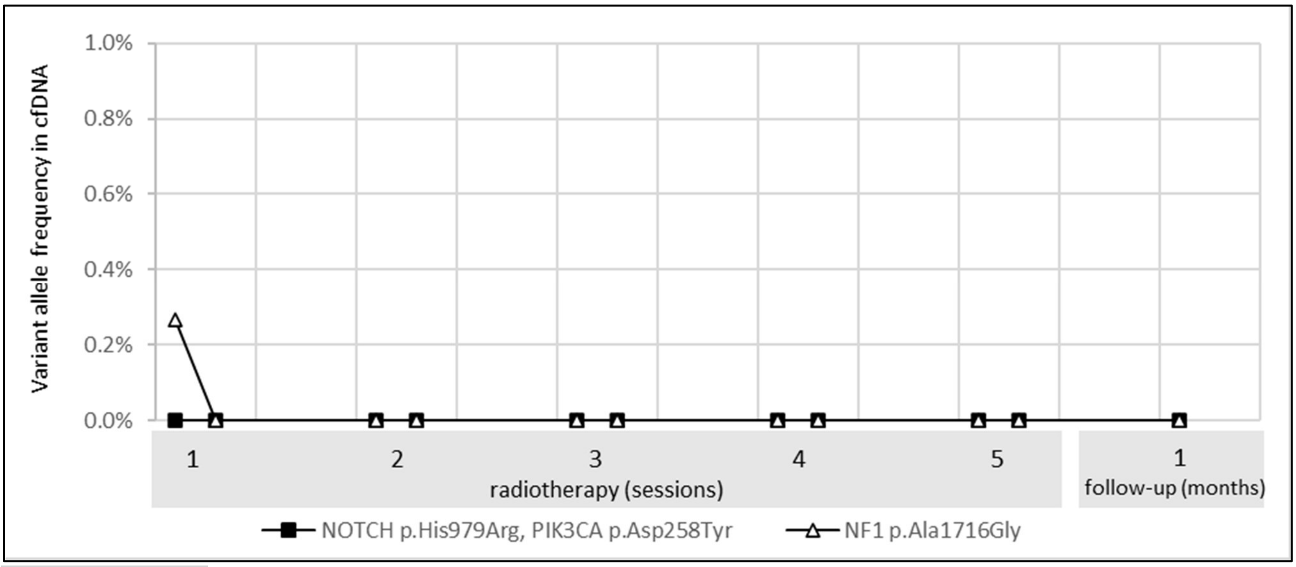

LOST TO FOLLOW-UP

- **Patient 20**

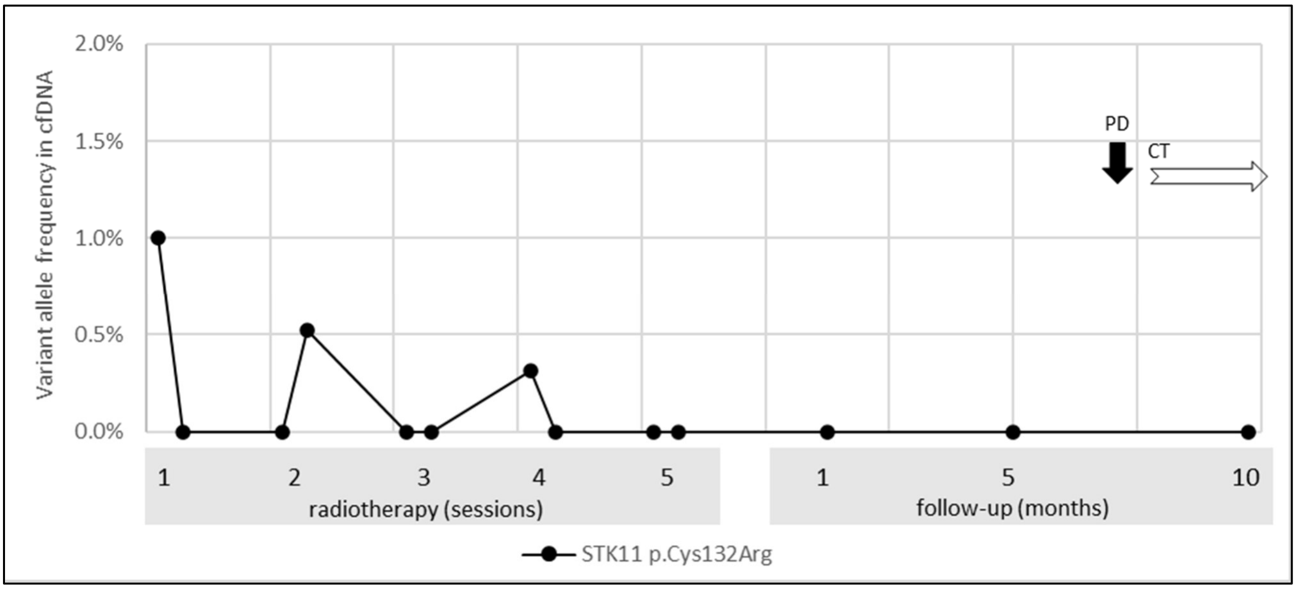

NO CONCORDANCE

- **Patient 23**

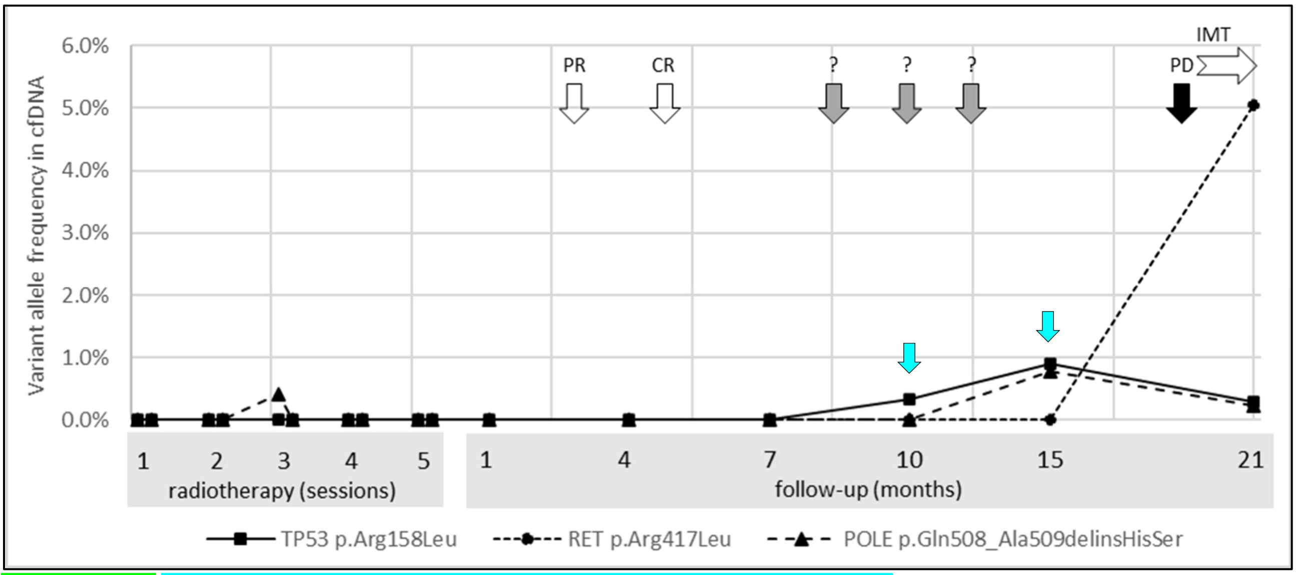

CONCORDANCE LIQUID BIOPSY ANTICIPATES RELAPSELIQUID BIOPSY ANTICIPATES RELAPSE

- **Patient 27**

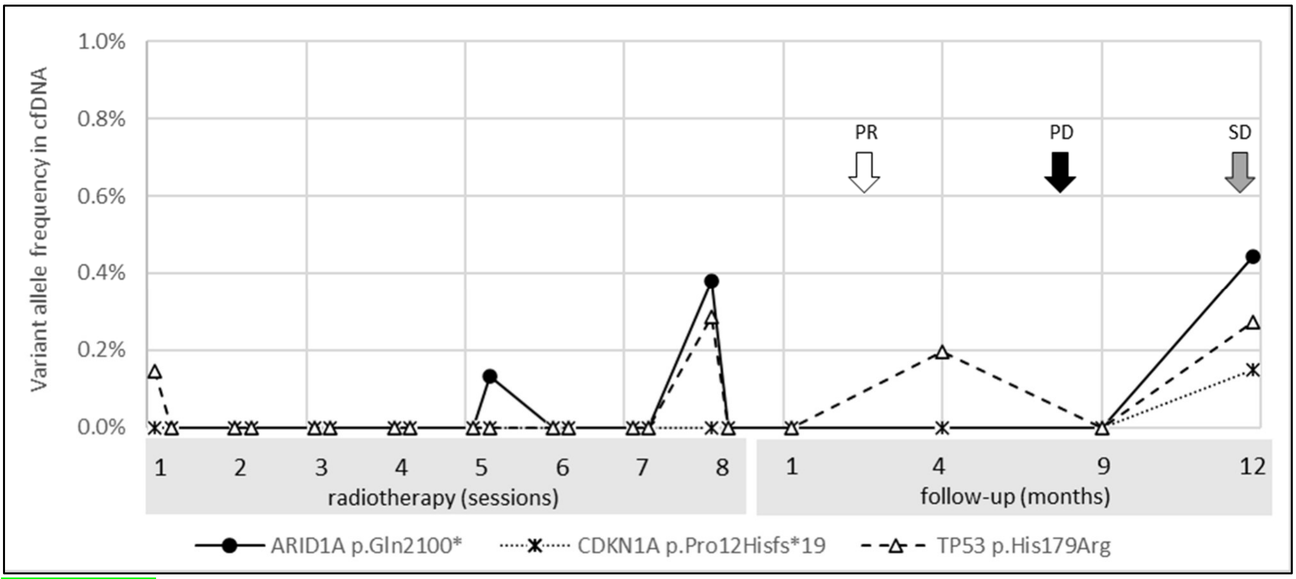

CONCORDANCE

- **Patient 28**

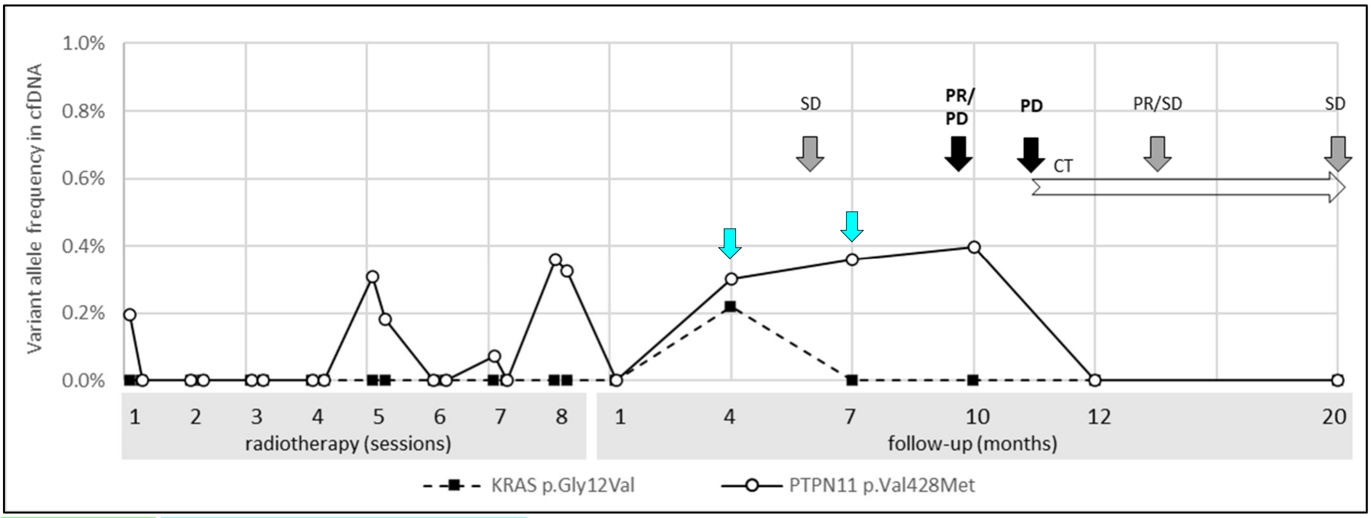

CONCORDANCE LIQUID BIOPSY ANTICIPATES RELAPSE

- **Patient 29**

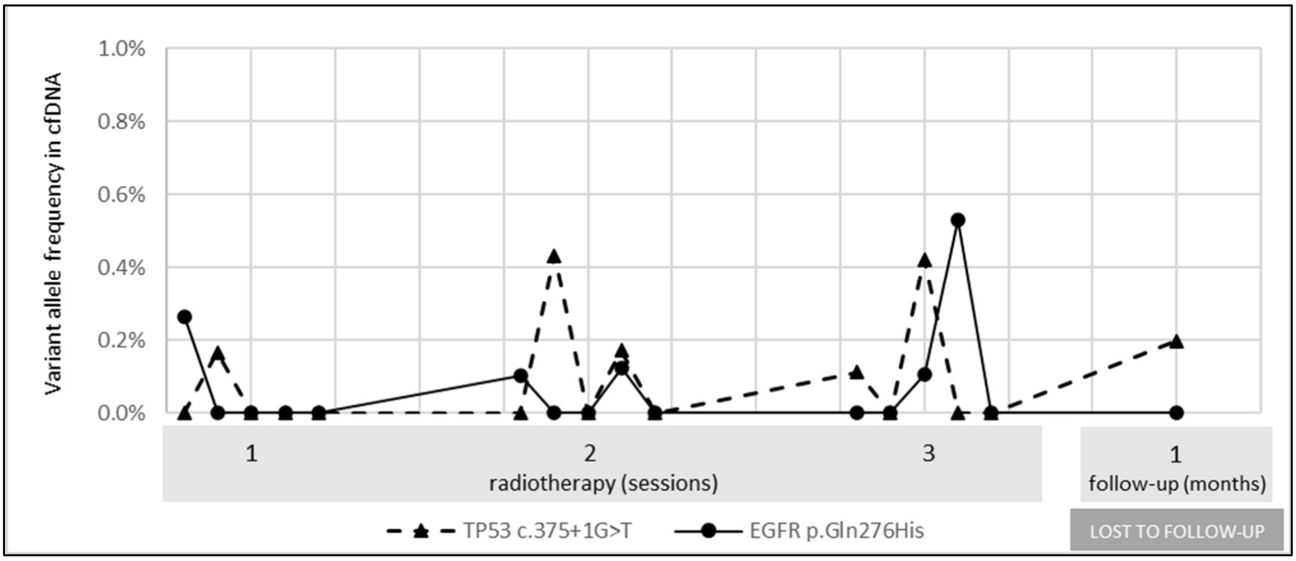

LOST TO FOLLOW-UP

- **Patient 30**

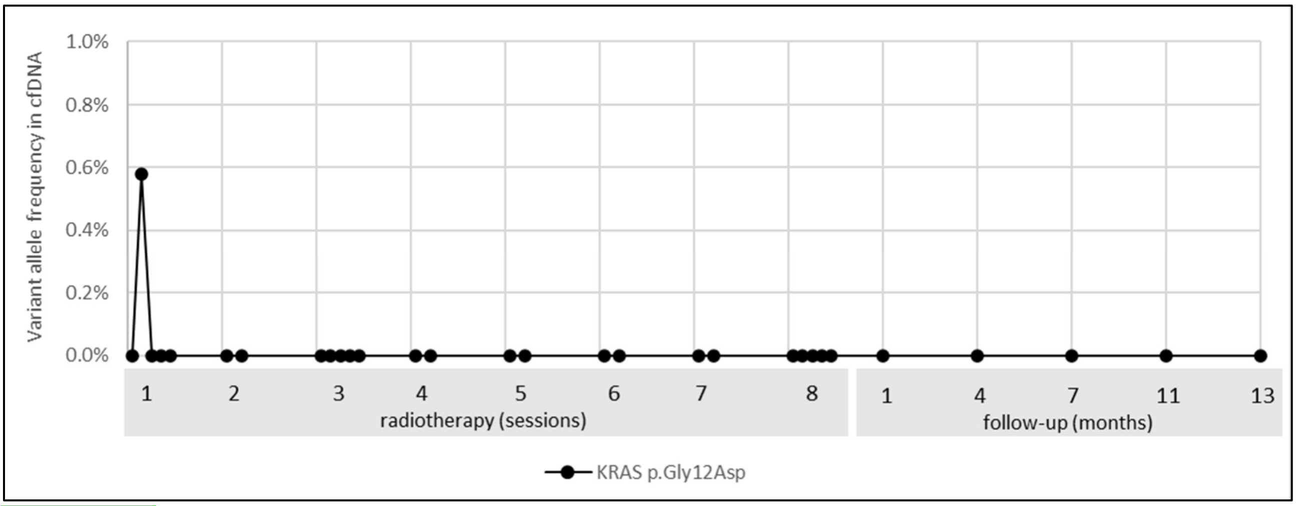

CONCORDANCE

- **Patient 32**

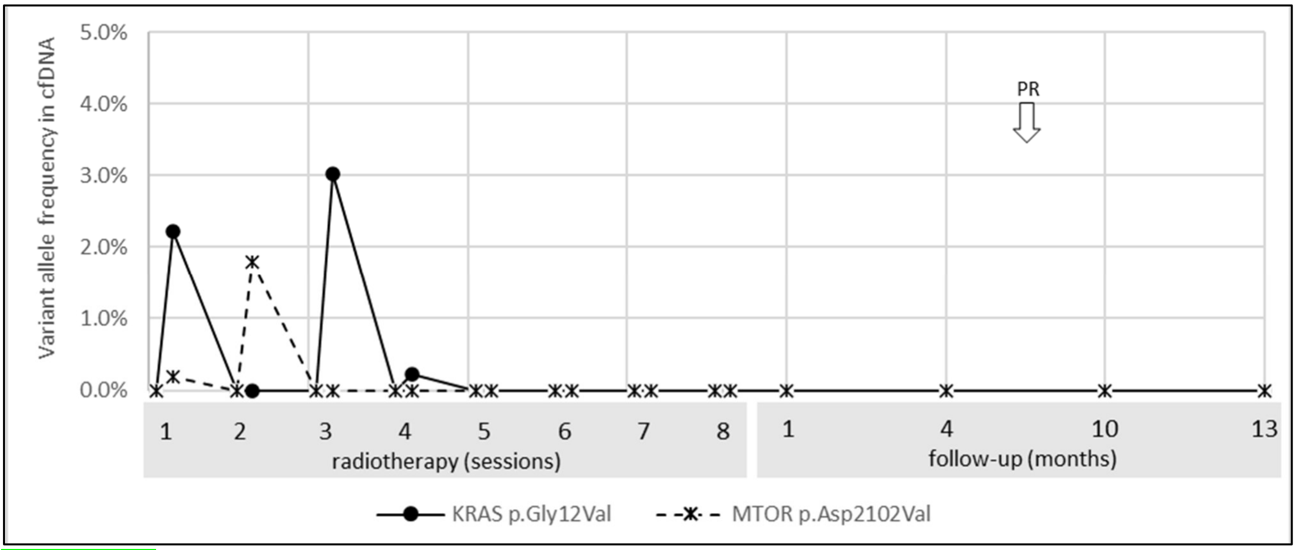

CONCORDANCE

- **Patient 33**

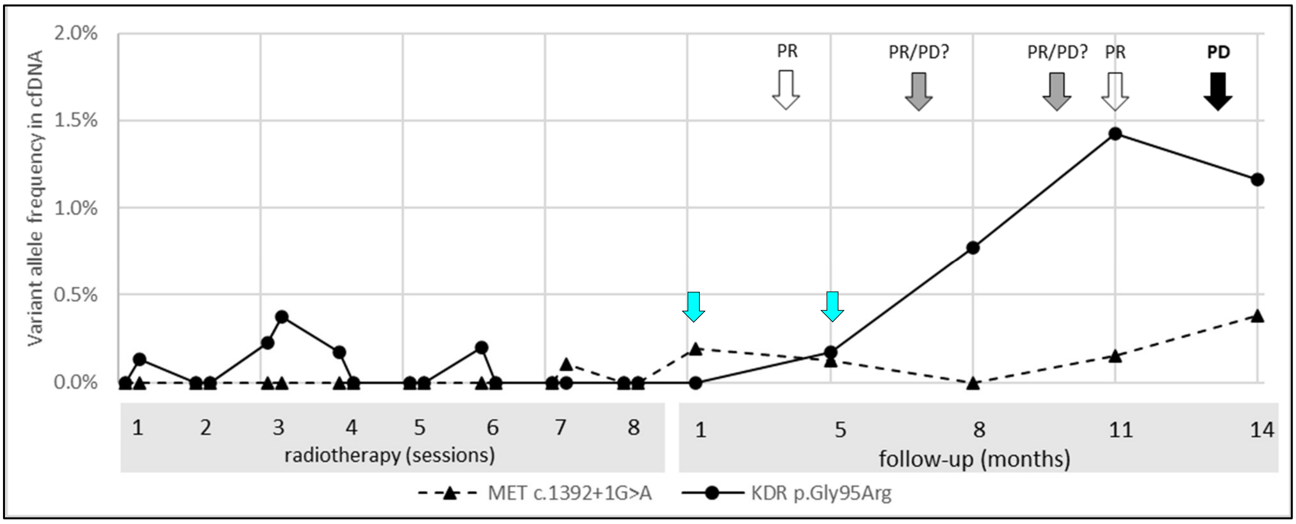

CONCORDANCE LIQUID BIOPSY ANTICIPATES RELAPSE

- **Patient 34**

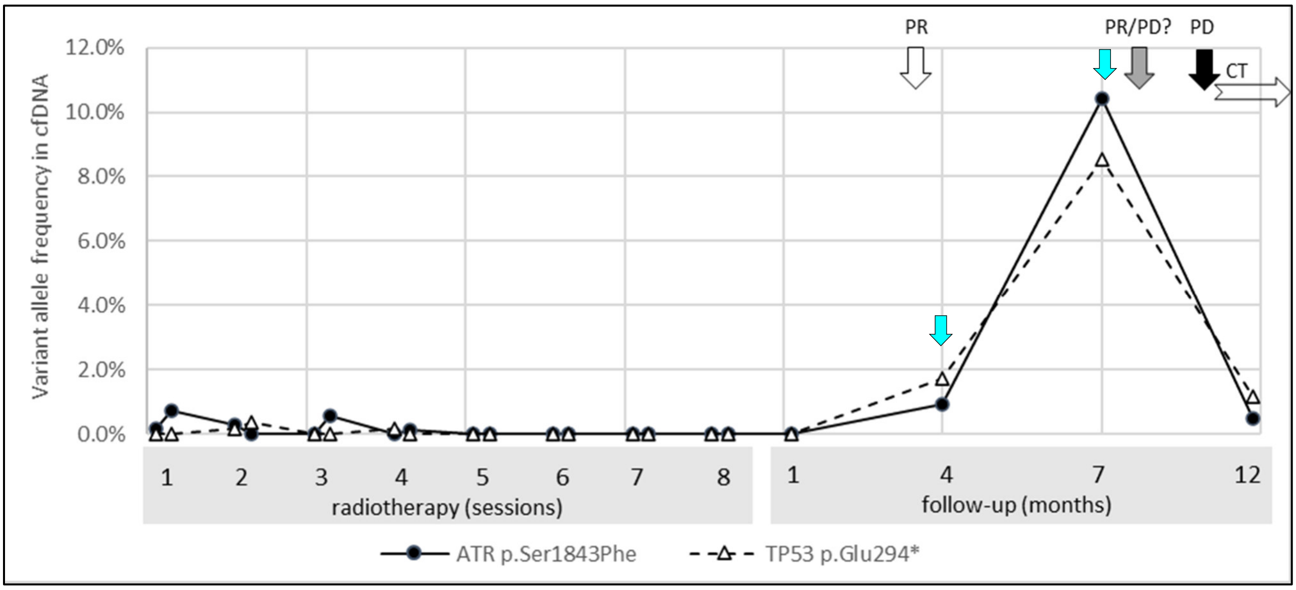

CONCORDANCE LIQUID BIOPSY ANTICIPATES RELAPSE

- **Patient 35**

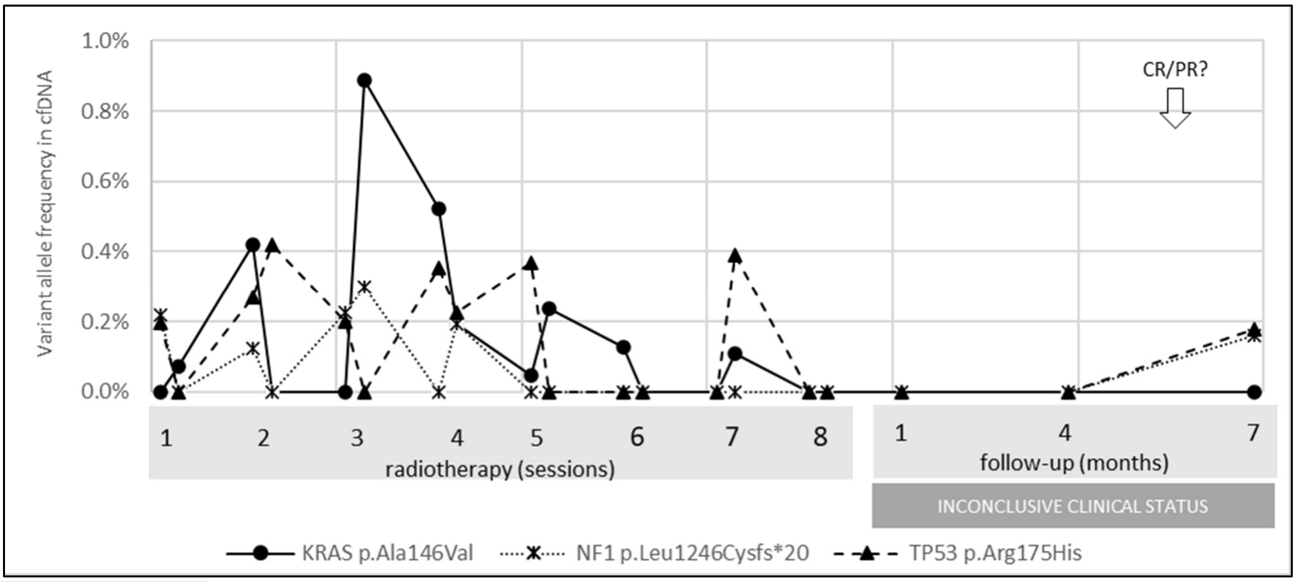

LOST TO FOLLOW-UP

- **Patient 36**

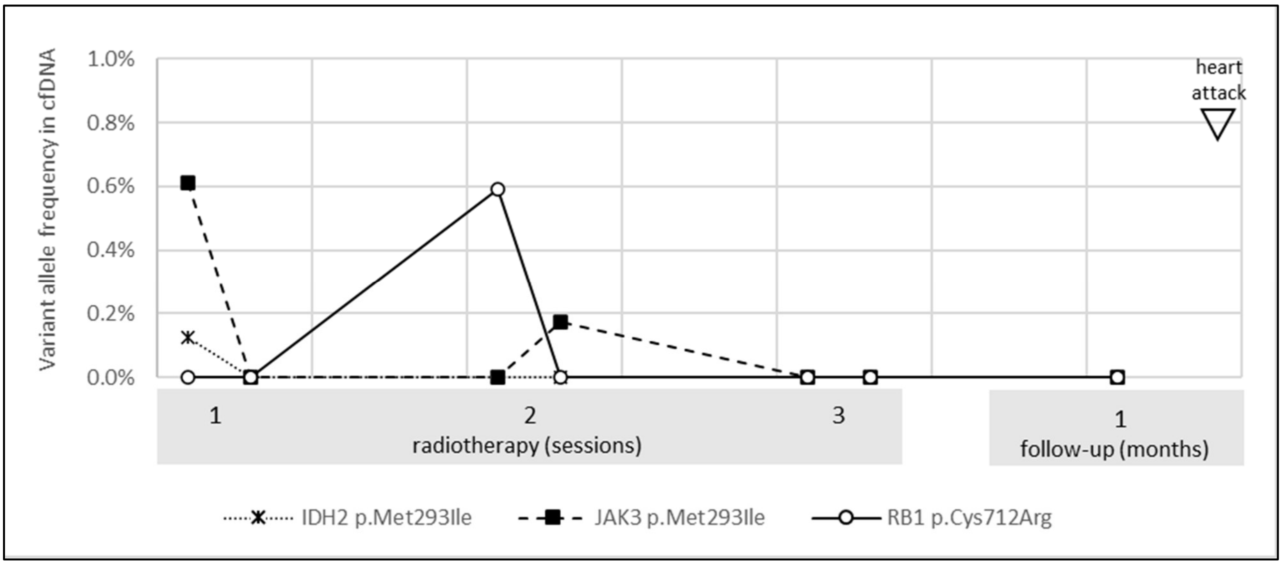

CONCORDANCE

- **Patient 37**

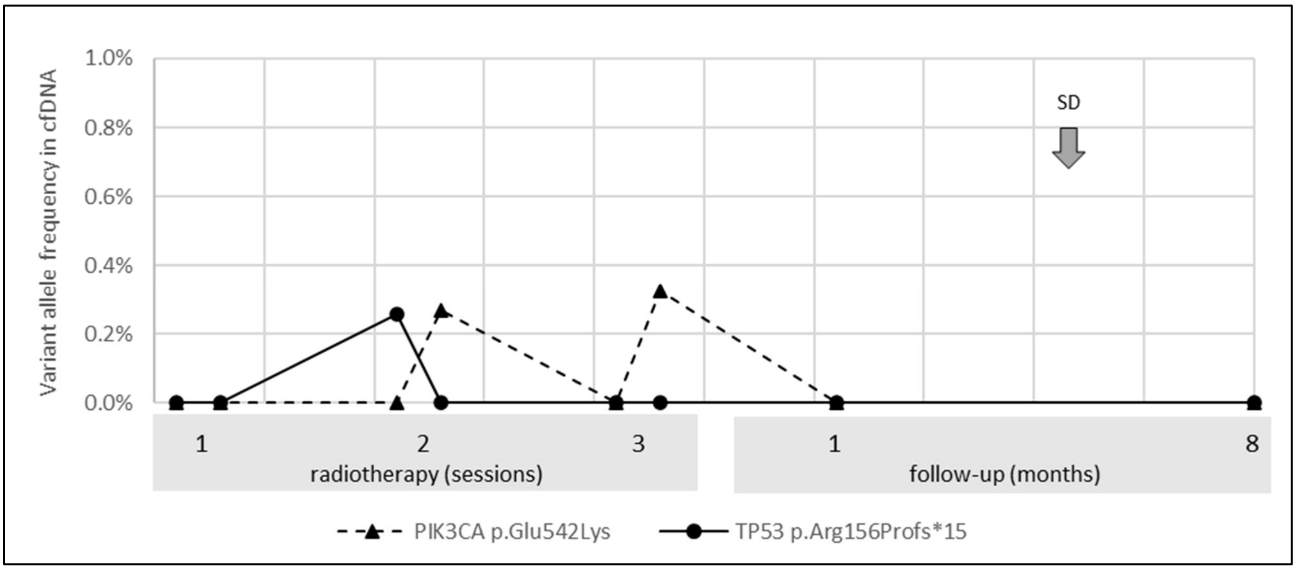

CONCORDANCE

- **Patient 39**

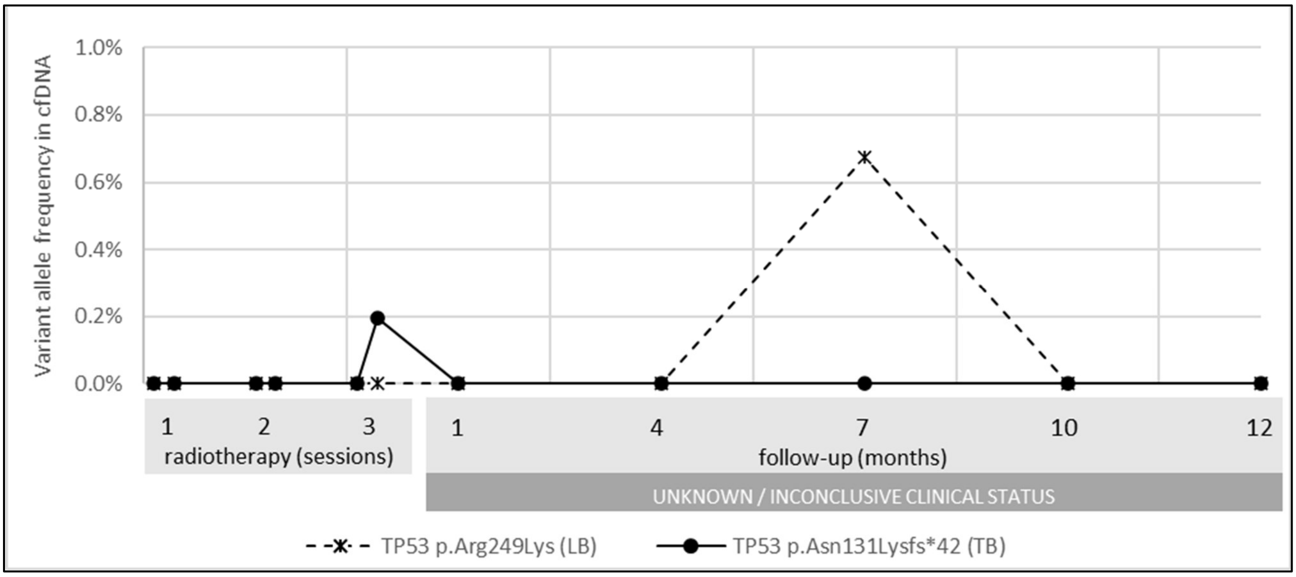

LOST TO FOLLOW-UP

- **Patient 40**

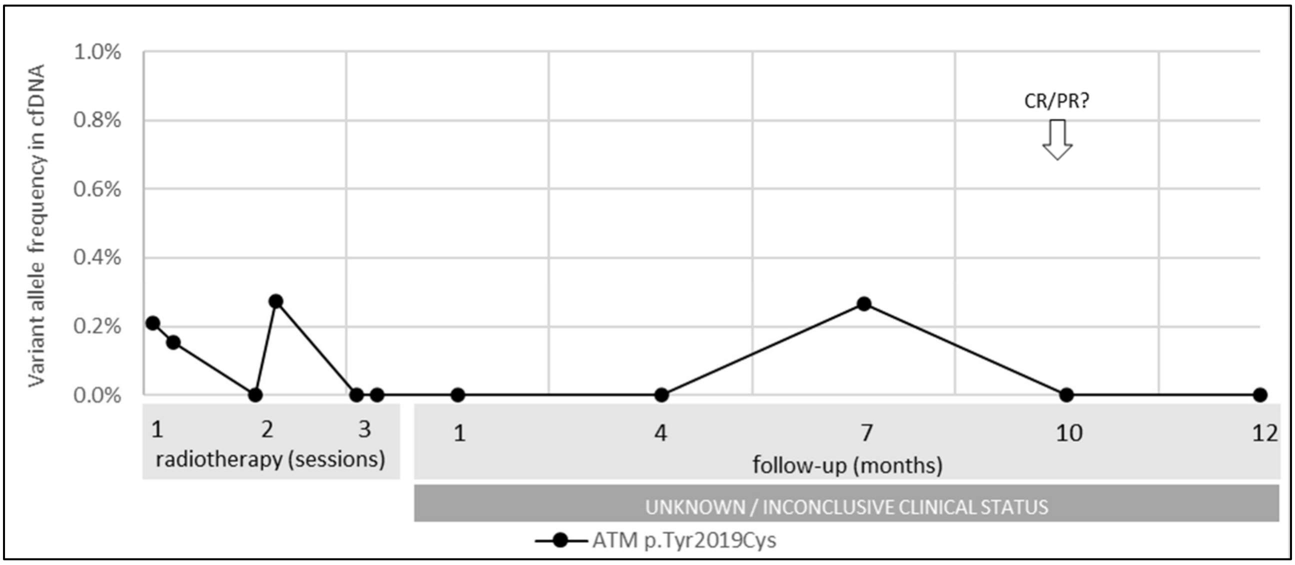

LOST TO FOLLOW-UP

- **Patient 42**

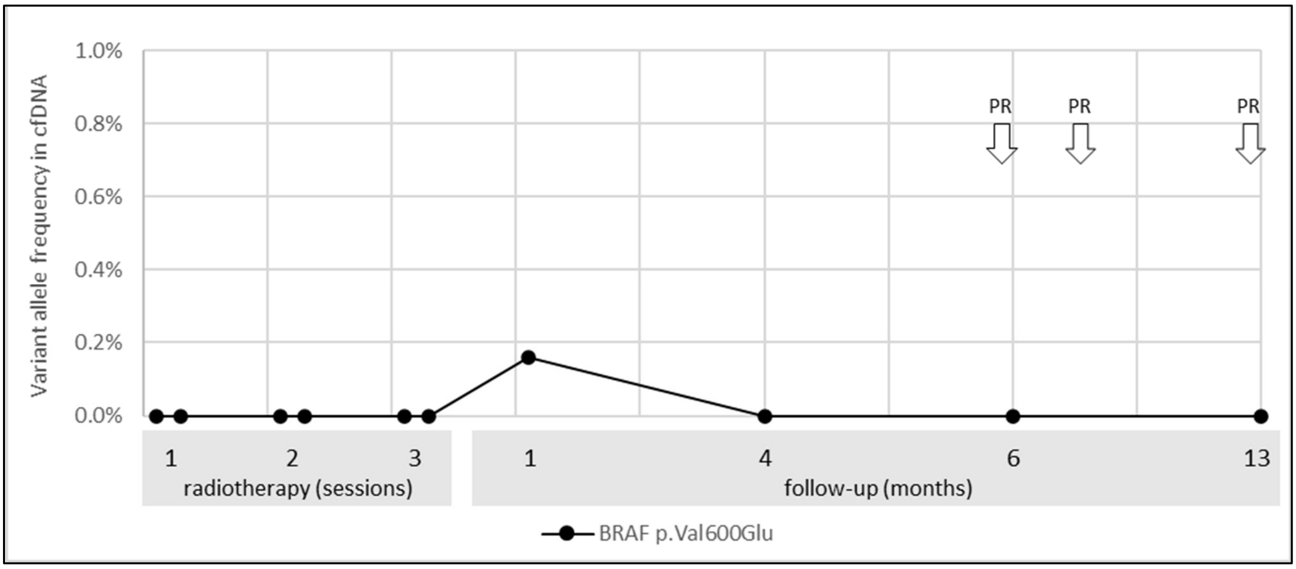

NO CONCORDANCE

- **Patient 43**

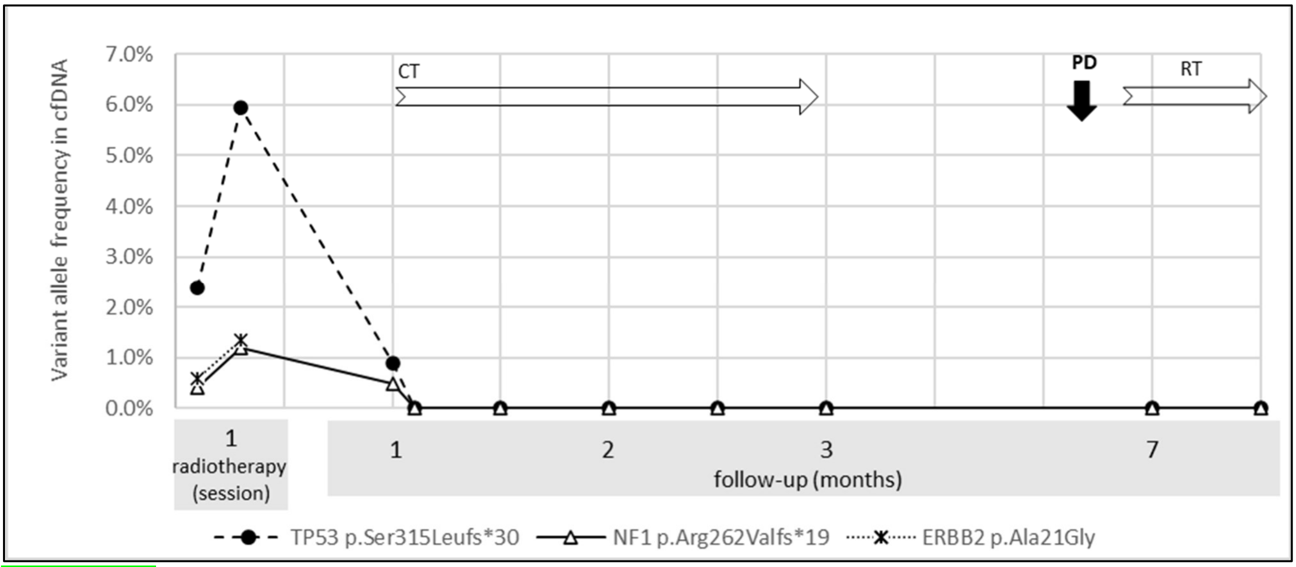

CONCORDANCE

- **Patient 44**

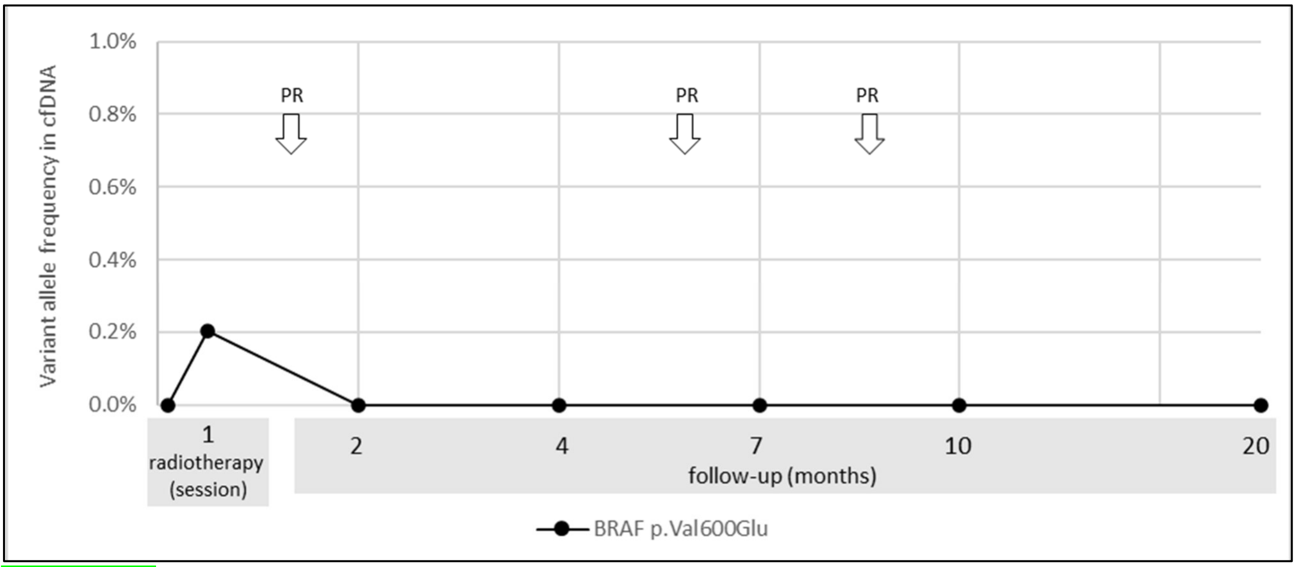

CONCORDANCE

- **Patient 45**

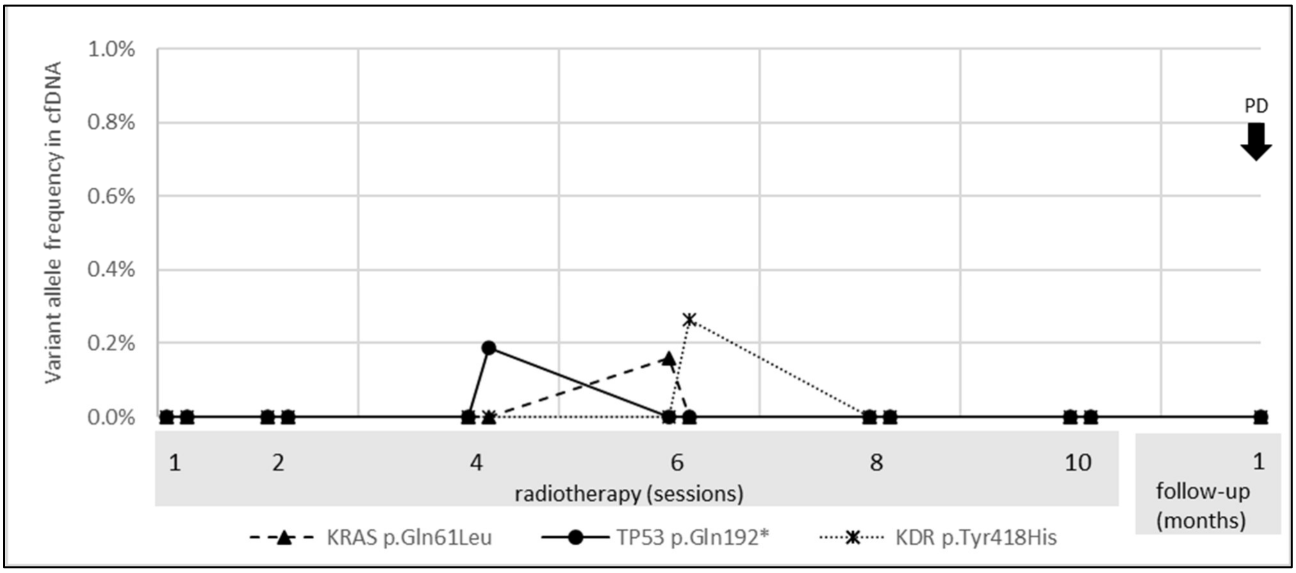

CONCORDANCE

- **Patient 48**

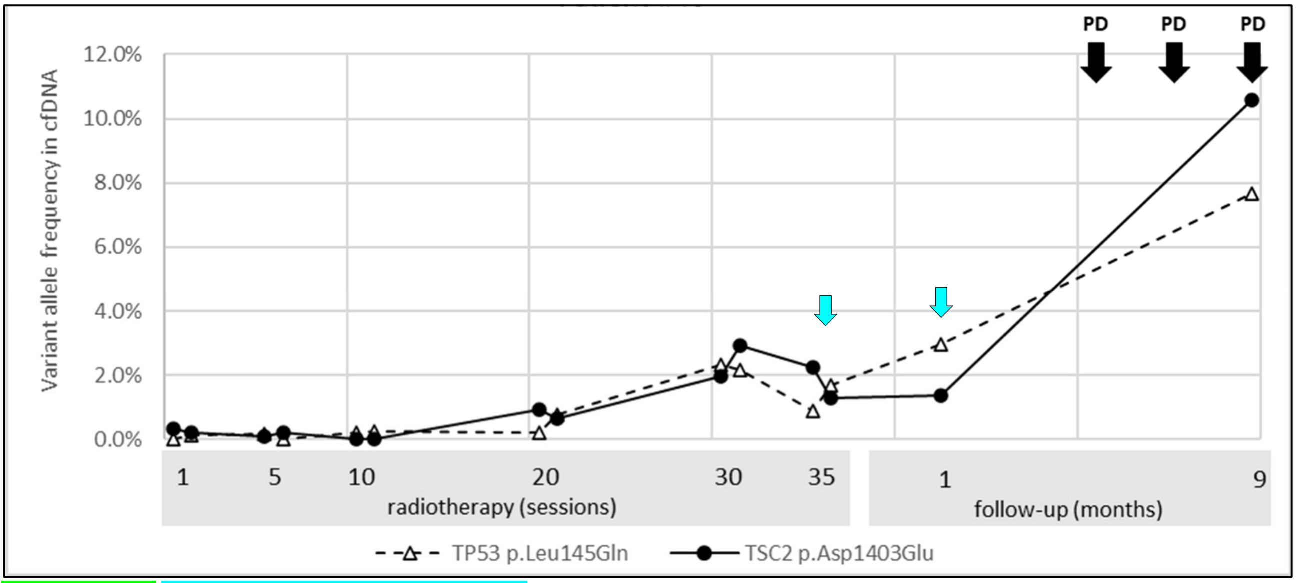

CONCORDANCE LIQUID BIOPSY ANTICIPATES RELAPSE

- **Patient 49**

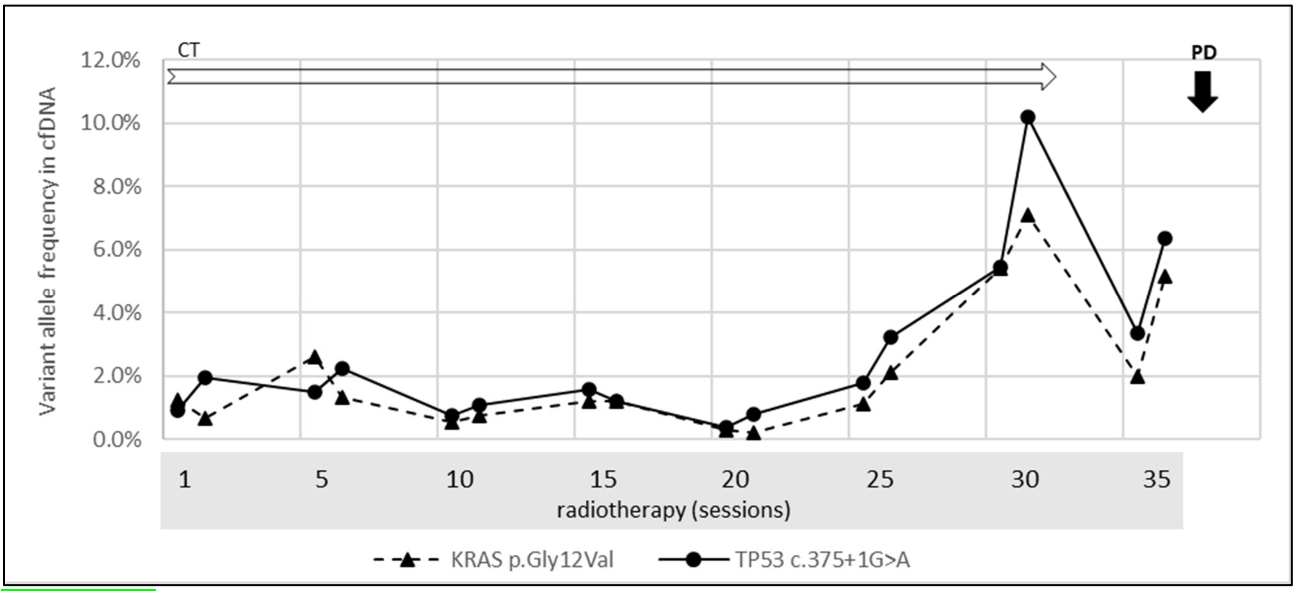

CONCORDANCE

- **Patient 50**

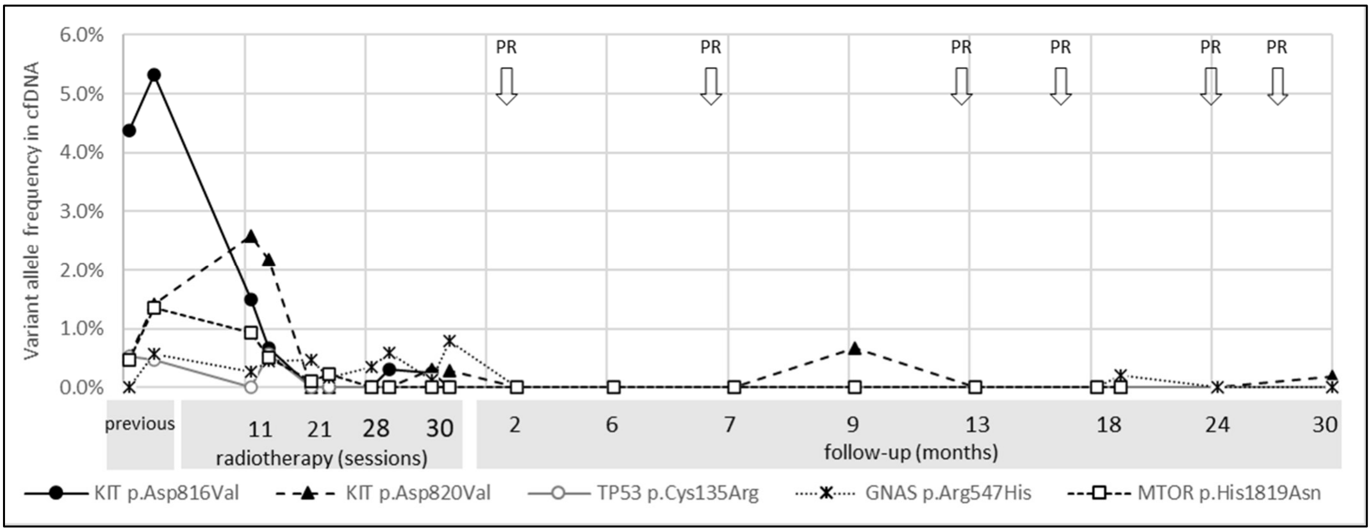

CONCORDANCE

Supplementary Figure 3. ***ctDNA dynamics and response assessment (according to RECIST criteria).***

The allelic frequency of selected tumour variants in serial liquid biopsies during the course of treatment and the follow-up is displayed for all the patients with longitudinal monitoring of ctDNA (excluding non-ctDNA shedders, with no detectable ctDNA levels). On the top, the response evaluation (RECIST criteria) is shown: a question mark (?) is included when the radiologic assessment is not completely conclusive, and an asterisk (*) is included when the assessment referred to other new lesions different from those irradiated during RT treatment. ctDNA signal and clinical status concordance conclusion is indicated below each graph. When a liquid biopsy and a diagnostic imaging test were performed more than five months apart (follow-up desynchronization), it is assumed that no conclusion about correlation can be taken, and the previous time points are considered for concordance evaluation (blue arrow). Liquid biopsy was considered to anticipate relapse when an increase in ctDNA signal is observed compared to previous liquid biopsy in two consecutive time points, at least for 1 biomarker (patients #23, #28, #33, #34 and #48).

RT, radiotherapy; CT, chemotherapy; IMT, immunotherapy; CR, complete response; PR, partial response; SD, stable disease; PD, progressive disease.

- **Patient 20**

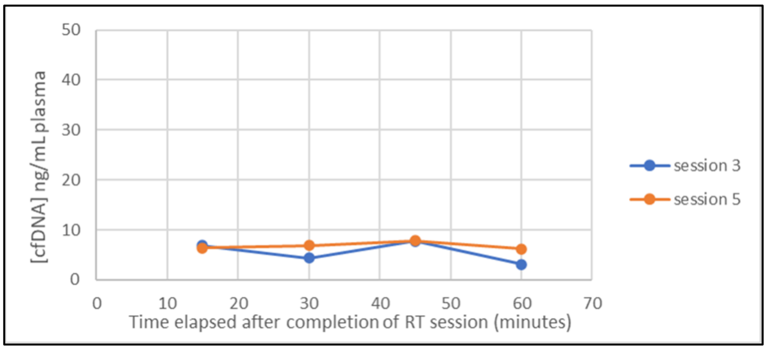

- **Patient 29**

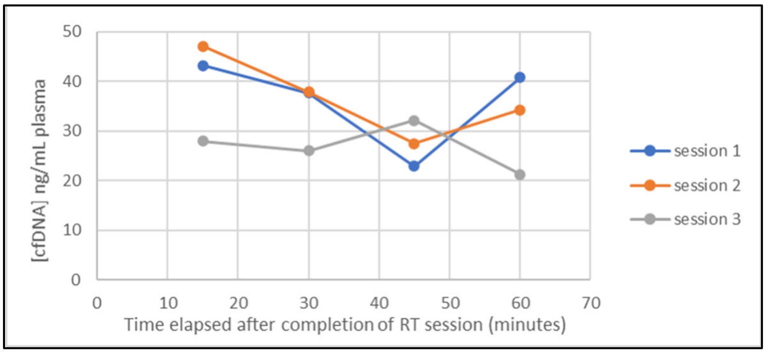

- **Patient 30**

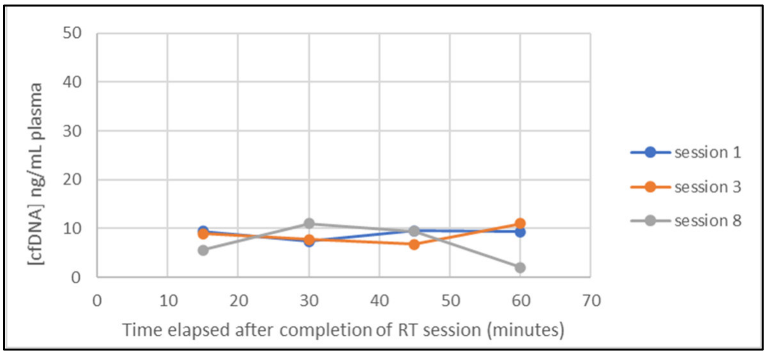

- **Patient 31**

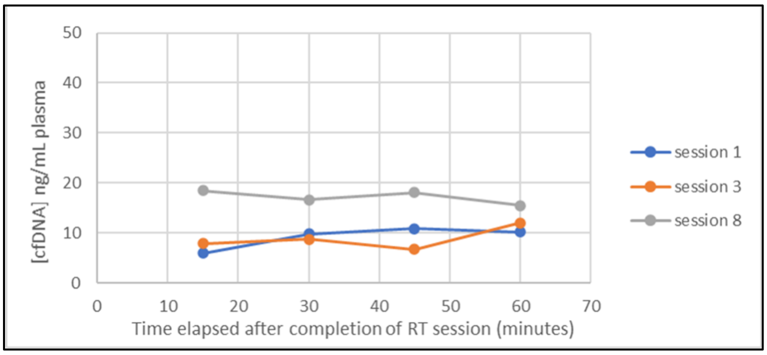

Supplementary Figure 4. ***[cfDNA] changes during RT in patients with multiple post-session liquid biopsies.***

[cfDNA] dynamics in the multiple liquid biopsies performed after specific RT sessions, at 15 minutes intervals. A wide variability was observed both between samples from different individuals and between samples from different RT sessions of the same individual.
